# Supplementary material for: Economy-wide evaluation of CO2 and air quality impacts of electrification in the United States
Source: Nat Commun. 2022 Nov 5;13:6693. doi: 10.1038/s41467-022-33902-9 (PMC9637153; doi:10.1038/s41467-022-33902-9)
Supplement: Supplementary file 1 — Supplementary Information [file 41467_2022_33902_MOESM1_ESM.pdf]

Supplemental Information for

Economy-Wide Evaluation of CO_2 and Air Quality Impacts of Electrification in the United States

John E.T. Bistline[a], Geoffrey Blanford[a], John Grant[b], Eladio Knipping[a], David L. McCollum[c], Uarporn Nopmongcol[b], Heidi Scarth[a], Tejas Shah[b], Greg Yarwood[b]

[a] Electric Power Research Institute, 3420 Hillview Avenue, Palo Alto, CA, 94304, USA
[b] Ramboll, 7250 Redwood Blvd., Suite 105, Novato, CA, 94945, USA
[c] Oak Ridge National Laboratory, 2360 Cherahala Blvd., Knoxville, TN 37932, USA

1. Overview of Methods

EPRI's U.S. Regional Economy, Greenhouse Gas, and Energy (US-REGEN) model finds electric sector and end-use pathways that meet demand given assumptions about policies, technologies, and markets. Regional aggregation for this analysis is (Supplementary Figure 1).

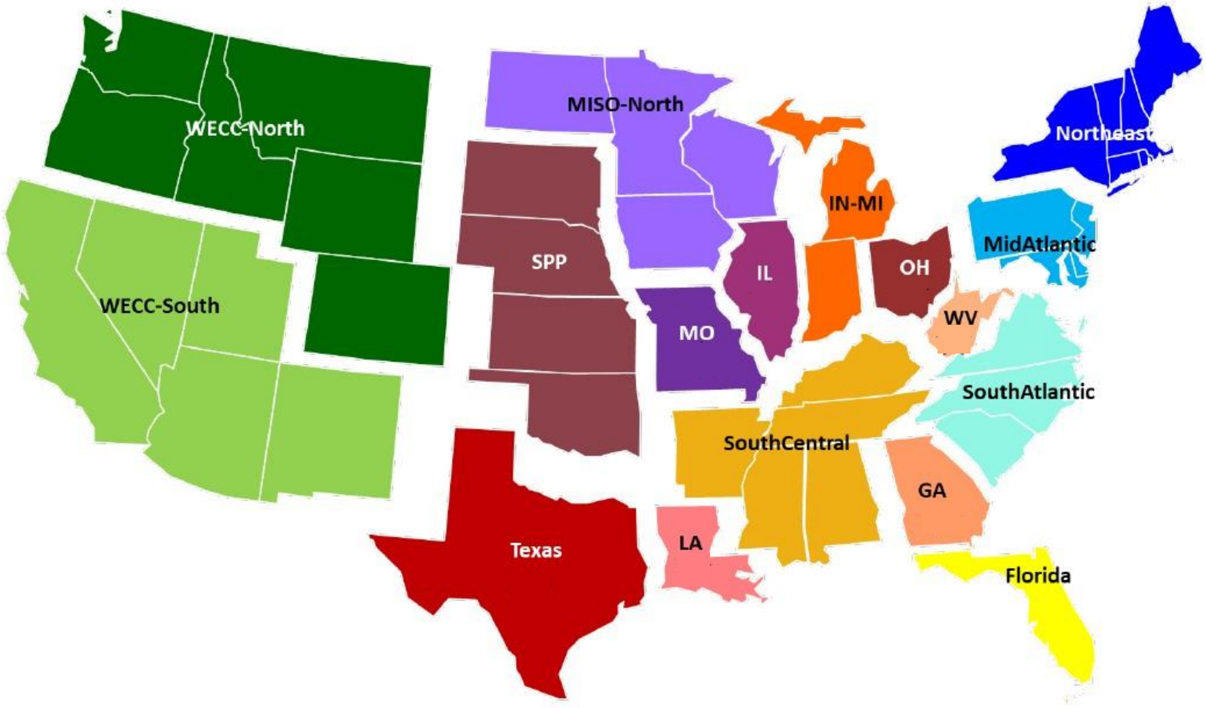

Supplementary Figure 1. Regional aggregation of US-REGEN for this analysis.

The electricity model includes endogenous capacity planning and dispatch with joint investment decisions for generation options, energy storage technologies, transmission, hydrogen production, and CO_2 removal, storage, and pipelines. Model decisions are made with perfect foresight.

The electric sector capacity expansion model is integrated with a technologically detailed consumer choice model of end-use service and energy demand, with the same geographic disaggregation and hourly temporal resolution. The end-use model represents the economic adoption of end-use technologies and fuels, particularly with respect to tradeoffs between electric and non-electric technologies. It includes a logit-based formulation for buildings and personal light-duty vehicle sectors to reflect behavioral heterogeneity by individual consumers in response to assumed total service costs of alternative technologies. In these sectors, the logit formulation is intended to represent behavioral heterogeneity rather than pure optimization. The market allocation in the logit model is still driven by assumed costs: The least-cost option does not capture 100% of the market allocation, but adoption rates are greater for technologies with greater cost advantage. The parameterization of the logit model determines the sensitivity of the market allocation (within a given nest in the nested structure) to assumed ("observed") costs, or conversely the influence of non-modeled ("non-observed") or costs or preferences, which by

construction are assumed to be independently and identically distributed for each technology in the nest. In the case of the buildings sectors, the model is primarily evaluating tradeoffs between electric heat pumps, electric resistance, and gas (or other non-electric) technologies. While the cost and performance of these technologies may evolve over time, they all exist today and have observable market shares that vary across regions of the U.S. and segments of the building stock. Thus, the choice of logit model parameters, which are not associated with technologies per se but rather with nests defining groups of technologies among which market share is allocated, can be informed by currently observed patterns.

In the case of light-duty vehicles, until very recently there has been essentially one technology deployed, namely the internal combustion engine vehicle (ICEV). With the rapid development and commercialization of electric vehicles over the past several years, adoption rates are growing, but current market shares cannot be construed as representing an equilibrium allocation given the very early stage of development and remaining barriers to scale. For this reason, the logit parameters in this light-duty vehicle sector are not informed by current data but are chosen to represent a hypothetical long-run equilibrium. Moreover, the model includes a lagged function assigned market allocation in a given time step as a linear combination of the logit model's allocation based on current costs and the previous time step's actual allocation. Thus, there is a gradual convergence to the outcomes of the logit formulation when those outcomes are significantly different than the current outcome. In practice, the modeled total costs of electric vehicles suggest a significant cost advantage over ICEVs even in the near-term, with the advantage increasing over time as battery costs fall further and potential policy incentives increase the relative costs of non-electric fuels. The result is increasing adoption based on economic fundamentals, the pace of which is not strongly impacted by the choice of logit parameters. More information about the formulation and the parameter choices is available in Chapter 3 of the model documentation.

For other sectors, including medium- and heavy-duty transport and industrial activities, technology tradeoffs are based on an optimization formulation. In all sectors, technology adoption evolves over time based on economic fundamentals such as improvements in cost and performance and scenario-based policy incentives. The end-use model also includes the supply of non-electric fuels, including upstream oil and gas and petroleum refining, based on a simplified representation that captures costs, energy inputs, and emissions drivers. The electricity and end-use models are run iteratively, with the electric model passing hourly electricity prices to the end-use model, and the end-use model passing back hourly load shapes and load growth, until energy prices and demands converge between the two models (Supplementary Figure 2).

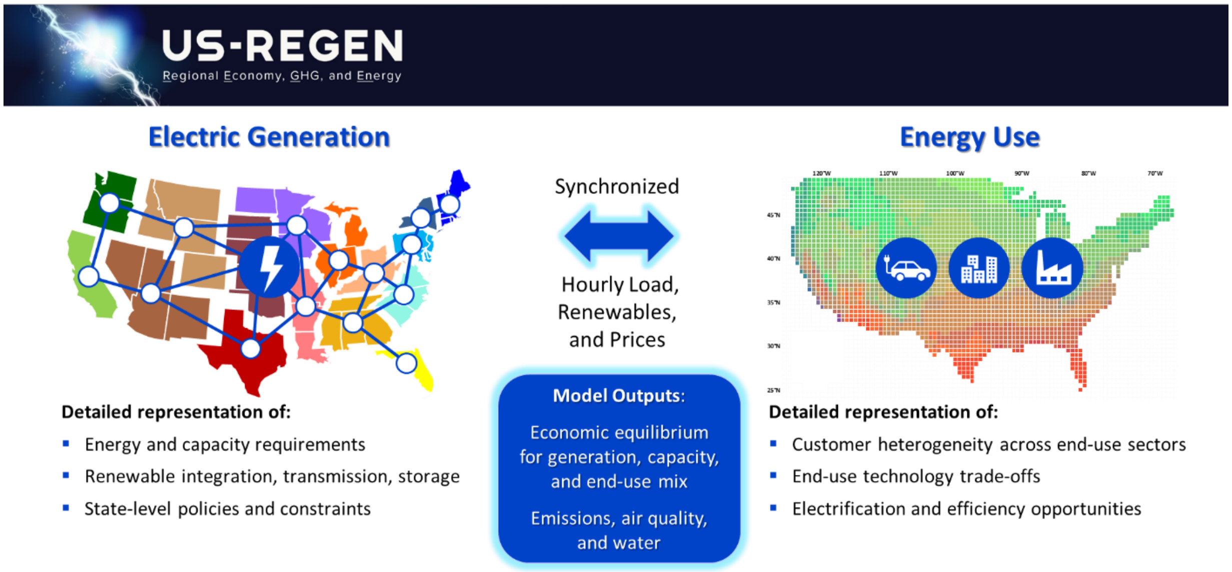

Supplementary Figure 2. Overview of the US-REGEN model. Full model documentation and other recent reports can be found at https://esca.epri.com/usregen.

The end-use model includes a separate module to evaluate rooftop solar PV adoption, investments in which are considered from the perspective of retail consumers, where installing behind-the-meter generation may offset retail purchases at flat volumetric rates. Rates are calculated using price outputs from the electric model and assume today's existing rate structures. The US-REGEN documentation Section 3.6 has more information (EPRI, 2020).

Electric vehicle charging shapes vary by household type, charging location, and day of the week and reflect a mix of charging locations (home, work, public) and power levels (Bistline, et al., 2021). Note that these shapes represent aggregate charging patterns rather than individual vehicle shapes. These charging profiles are based on research by EPRI's Electric Transportation program and come from a combination of aggregated empirical data and simulation. REGEN also provides options for flexible charging, including for a fleet of autonomous vehicles. The participation share in charging flexibility programs is assumed to be 50% for residential households and 80% for workplace charging. The flexible charging module in REGEN minimizes the cost of charging subject to constraints that the daily vehicle energy requirement for each profile must be met in the available hours and that charging in each hour is limited by available capacity. The focus of the charging module is load shifting, where vehicle charging shifts in time as response to utility control, time-varying pricing, or other incentives.

Hourly profiles used in the model solution are based on a single representative year (2015 for these experiments), and the same underlying meteorology and temperatures are used in the end-use model to develop hourly load shapes (e.g., for electric space heating in residential and commercial buildings) to avoid dampening variance through multi-year averaging. While consideration of multiple weather years may reveal more extreme events, there are significant wind droughts observed in the sample year, reinforcing the importance of energy storage and firm resources for balancing. Moreover, the model includes a reserve requirement that firm capacity exceed the residual peak load in each region, suggesting the results are relatively robust to extended wind and solar droughts. Additional detail on wind and solar resource assumptions and technology characteristics is provided in Section 2.4 of the REGEN documentation (EPRI, 2020).

2. Development of Emissions Input for Air Quality Modeling

US-REGEN emission estimates are projected from the 2014 National Emissions Inventory (NEI; 2014v7.1 Platform[1]) to future years based on scenario-specific changes to 2015–2050 economic activity, technology, and emissions controls. The effects of emission control programs on future emission inventory forecasts were estimated for on-road vehicles (MOVES2014b and EMFAC2017), off-road[2] equipment (MOVES2014b), rail and harbor craft (Final Rule for Control of Emissions of Air Pollution From Locomotive Engines and Marine Compression-Ignition Engines Less Than 30 Liters per Cylinder, EPA, 2008), and commercial marine vessels (Final Rule for Control of Emissions From New Marine Compression-Ignition Engines at or Above 30 Liters per Cylinder (EPA, 2009).

US-REGEN future-year emissions were transformed into emission inventory inputs for use in air quality modeling. US-REGEN sector, source, fuel type, and region were cross-referenced to source category classification code (SCC) and county for nonpoint sources and SCC, county, and North American Industry Classification System (NAICS) for point sources. US-REGEN emissions were then mapped into air quality modeling format based on 2014 NEI emissions. For US-REGEN emissions that comprised multiple 2014 NEI categories, emissions were disaggregated in accordance with 2014 NEI distributions (e.g., by county) while US-REGEN emission totals were retained (e.g., statewide).

Future-year emissions were further adjusted to improve consistency with the 2016 baseline (2016 EI). Although the EPA's 2014 (US-REGEN emission baseline) and 2016 EI (air quality modeling baseline) are mostly comparable, there are notable differences in oil & gas, commercial marine vessel Class 1&2 and Class 3, pleasure craft, and rail due to changing methodology or underlying data. For these sectors, US-REGEN "forecast factors" (e.g., the relative changes in emissions from 2035 vs 2015) were applied to the 2016 EI. Certain non-electrified sources including fugitive dust (except for paved road dust), residential wood combustion, and agricultural burning are held constant from the 2016 EI with an assumption that future controls will offset growth.

The US-REGEN emissions were processed to develop more temporally, spatially, and chemically refined inventory inputs required for air quality modeling using the SMOKE system[3]. The emissions were processed by EPA standard source categorization, i.e., emission sector. This is done to assist in the quality assurance of the emissions modeling by comparing emissions developed in this study with the EPA's 2016 EI:

- **Agricultural**: Agricultural fertilizer and livestock emissions
- **Residential wood combustion (RWC)**: Residential wood-burning devices such as fireplaces and wood stoves
- **Oil & Gas**: Oil and gas exploration and production sources
- **Fugitive dust**: Fugitive dust sources such as paved/unpaved road dust and construction dust

---

[1] https://www.epa.gov/air-emissions-modeling/2014-version-71-platform; last accessed on October 14, 2021

[2] Note that "off-road" or sometimes "non-road" refers to mobile sources other than on-road vehicles (i.e., cars, trucks, buses), which includes aviation, rail, maritime, as well as non-road surface vehicles used in agriculture, construction, and mining.

[3] http://smoke-model.org/

- **Other Nonpoint**: Stationary nonpoint sources including industrial/ commercial/residential fuel combustion, solvent utilization for surface coating and cleaning/degreasing, commercial cooking, and waste disposal
- **CMV Class 1&2**: Category 1 and 2 Commercial Marine Vessels (CMV C1&C2)
- **Rail**: Locomotives
- **Nonroad mobile**: Non-road mobile sources such as non-road construction, industrial, and lawn and garden equipment
- **On-road mobile**: On-road mobile sources, such as light-duty passenger vehicles and heavy-duty trucks
- **Non-EGU point**: Industrial point sources excluding power plants
- **EGU**: Electric generating units

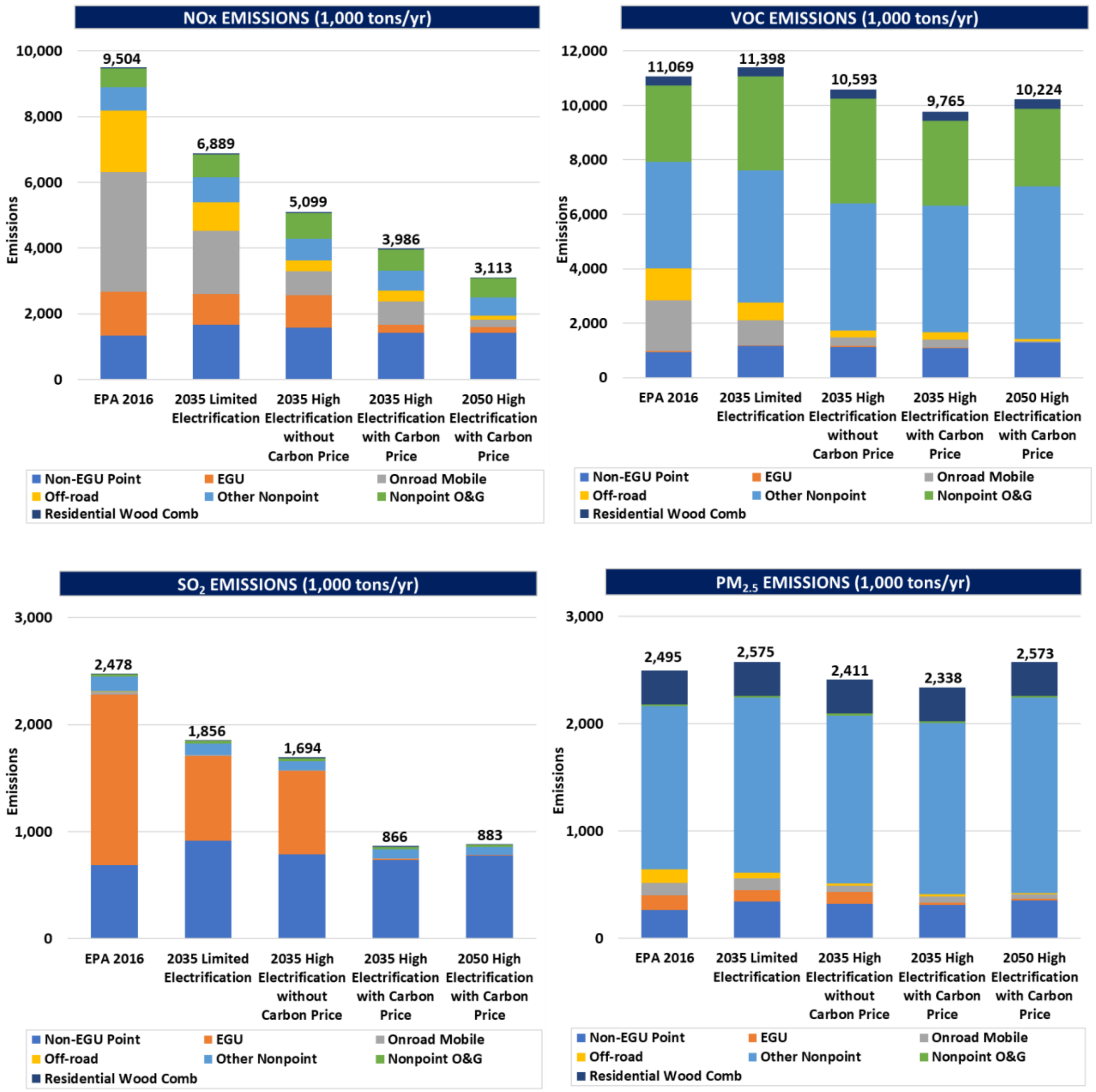

Supplementary Figure 3. CONUS NOx (top left), VOC (top right), SO_2 (bottom left), $PM_{2.5}$ (bottom right) emissions in thousand tons per year by source category.

Note that changes in the primary PM emissions in the other nonpoint emissions are driven in large part by changes in two activities: cooking and wood combustion. Cooking emissions increase steadily over time due to increases in service demand (even with electrification trends). Wood combustion changes from the Limited Electrification scenario to the High Electrification scenario without a carbon price, as there is less wood consumption due to increased electrification. However, when a carbon price is added, there is an increased use of biomass because it is a low-carbon fuel and appears relatively cheaper than electrification for certain households and climate zones. This effect is happening primarily in buildings due to space heating.

Supplementary Table 1. Emissions in thousand tons per year by source category.

Scenario	Pollutant	Non-EGU Point	EGU	Onroad Mobile	Nonroad Mobile	Rail	CMV Class 1&2	Other Nonpoint	Nonpoint O&G	Residential Wood Comb	Agricultural	Anthro Fugitive Dust	Total
EPA 2016	CO	1,657	661	19,997	10,720	105	30	2,649	760	2,120	0	0	38,698
2035 Limited Electrification	CO	2,510	857	7,505	7,552	69	20	3,151	937	2,120	0	0	24,719
2035 High Electrification without Carbon Price	CO	2,384	938	2,561	2,919	27	8	3,078	1,051	2,120	0	0	15,086
2035 High Electrification with Carbon Price	CO	2,273	638	2,481	2,906	24	8	3,084	848	2,120	0	0	14,381
2050 High Electrification with Carbon Price	CO	2,436	454	348	1,073	10	3	3,501	777	2,120	0	0	10,721
EPA 2016	NH_3	68	24	101	2	0	0	79	0	15	3,410	0	3,699
2035 Limited Electrification	NH_3	95	49	76	1	0	0	139	0	15	4,126	0	4,501
2035 High Electrification without Carbon Price	NH_3	93	54	26	1	0	0	128	0	15	4,126	0	4,444
2035 High Electrification with Carbon Price	NH_3	91	33	25	1	0	0	125	0	15	4,126	0	4,416
2050 High Electrification with Carbon Price	NH_3	103	26	3	0	0	0	127	0	15	4,603	0	4,878
EPA 2016	NOx	1,330	1,338	3,641	1,117	559	204	712	572	31	0	0	9,504
2035 Limited Electrification	NOx	1,674	931	1,920	379	363	136	750	705	31	0	0	6,889
2035 High Electrification without Carbon Price	NOx	1,585	981	725	148	143	52	642	791	31	0	0	5,099
2035 High Electrification with Carbon Price	NOx	1,423	241	719	146	126	52	609	638	31	0	0	3,986
2050 High Electrification with Carbon Price	NOx	1,423	173	227	49	51	19	554	585	31	0	0	3,113
EPA 2016	PM_{10}	409	166	241	110	16	6	574	14	318	0	7,748	9,602
2035 Limited Electrification	PM_{10}	521	132	286	45	11	4	703	17	317	0	7,440	9,476
2035 High Electrification without Carbon Price	PM_{10}	497	135	222	17	4	1	673	19	317	0	7,086	8,972
2035 High Electrification with Carbon Price	PM_{10}	470	24	222	17	4	1	707	16	317	0	7,086	8,864
2050 High Electrification with Carbon Price	PM_{10}	549	15	225	7	2	1	941	14	317	0	7,199	9,269

EPA 2016	$PM_{2.5}$	266	135	118	104	16	5	467	14	317	0	1,053	2,495
2035 Limited Electrification	$PM_{2.5}$	341	106	111	41	10	4	582	17	317	0	1,046	2,575
2035 High Electrification without Carbon Price	$PM_{2.5}$	322	108	61	16	4	1	563	19	317	0	999	2,411
2035 High Electrification with Carbon Price	$PM_{2.5}$	310	22	60	16	4	1	593	16	317	0	999	2,338
2050 High Electrification with Carbon Price	$PM_{2.5}$	355	13	44	7	1	0	796	14	317	0	1,025	2,573
EPA 2016	SO_2	690	1,592	28	2	0	1	139	19	8	0	0	2,478
2035 Limited Electrification	SO_2	918	786	11	1	0	0	108	24	8	0	0	1,856
2035 High Electrification without Carbon Price	SO_2	786	782	4	1	0	0	87	27	8	0	0	1,694
2035 High Electrification with Carbon Price	SO_2	737	11	4	1	0	0	85	21	8	0	0	866
2050 High Electrification with Carbon Price	SO_2	777	5	1	0	0	0	73	20	8	0	0	883
EPA 2016	VOC	946	34	1,862	1,140	26	8	3,725	2,792	341	195	0	11,069
2035 Limited Electrification	VOC	1,168	26	906	641	17	5	4,618	3,441	341	236	0	11,398
2035 High Electrification without Carbon Price	VOC	1,133	28	314	247	7	2	4,423	3,863	341	236	0	10,593
2035 High Electrification with Carbon Price	VOC	1,092	11	304	246	6	2	4,413	3,115	341	236	0	9,765
2050 High Electrification with Carbon Price	VOC	1,287	8	36	91	2	1	5,342	2,854	341	263	0	10,224

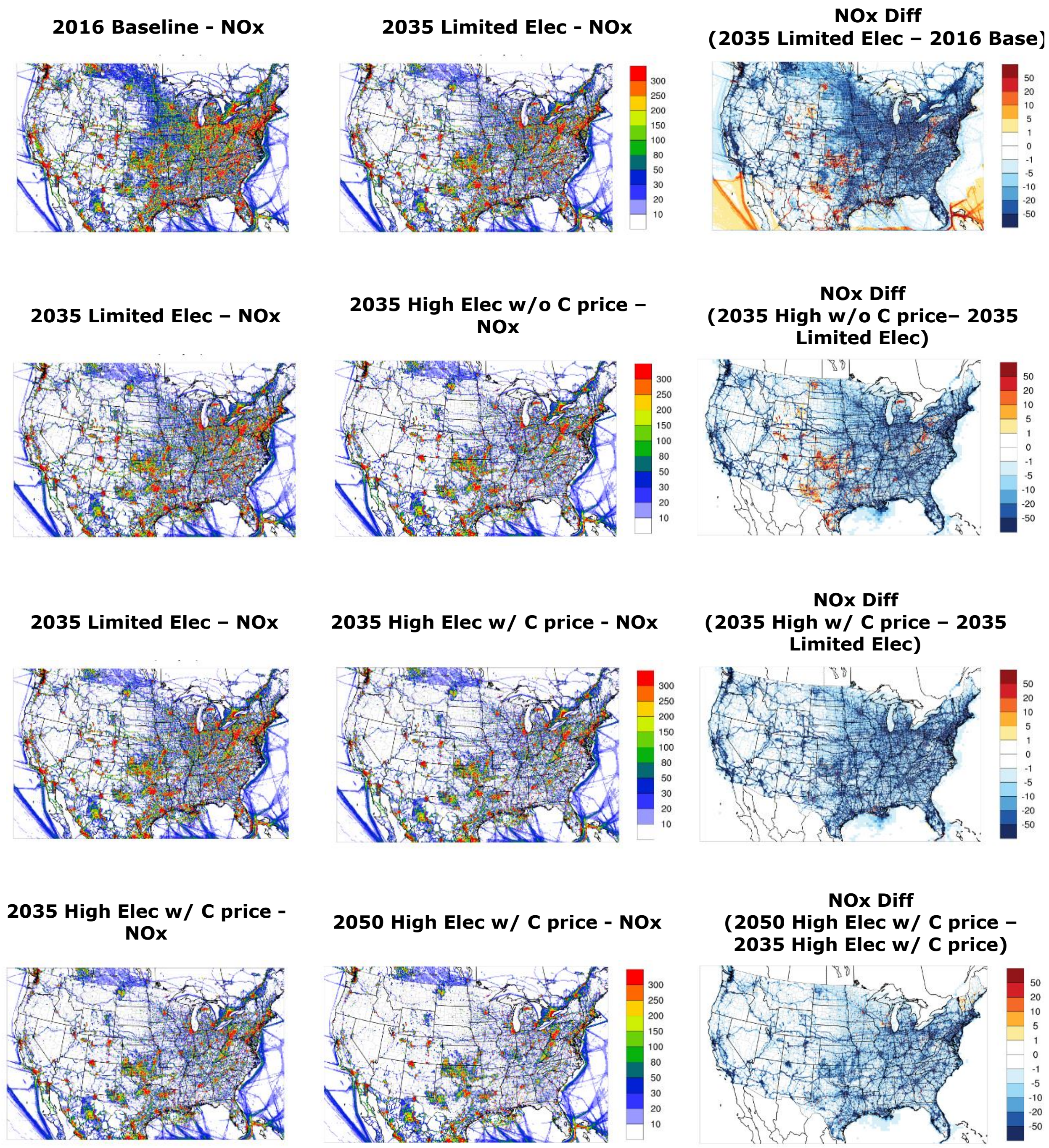

Supplementary Figure 4. Spatial distribution of NOx emissions (tons per year) for the CONUS and emissions difference map between Electrification with Carbon Price scenarios.

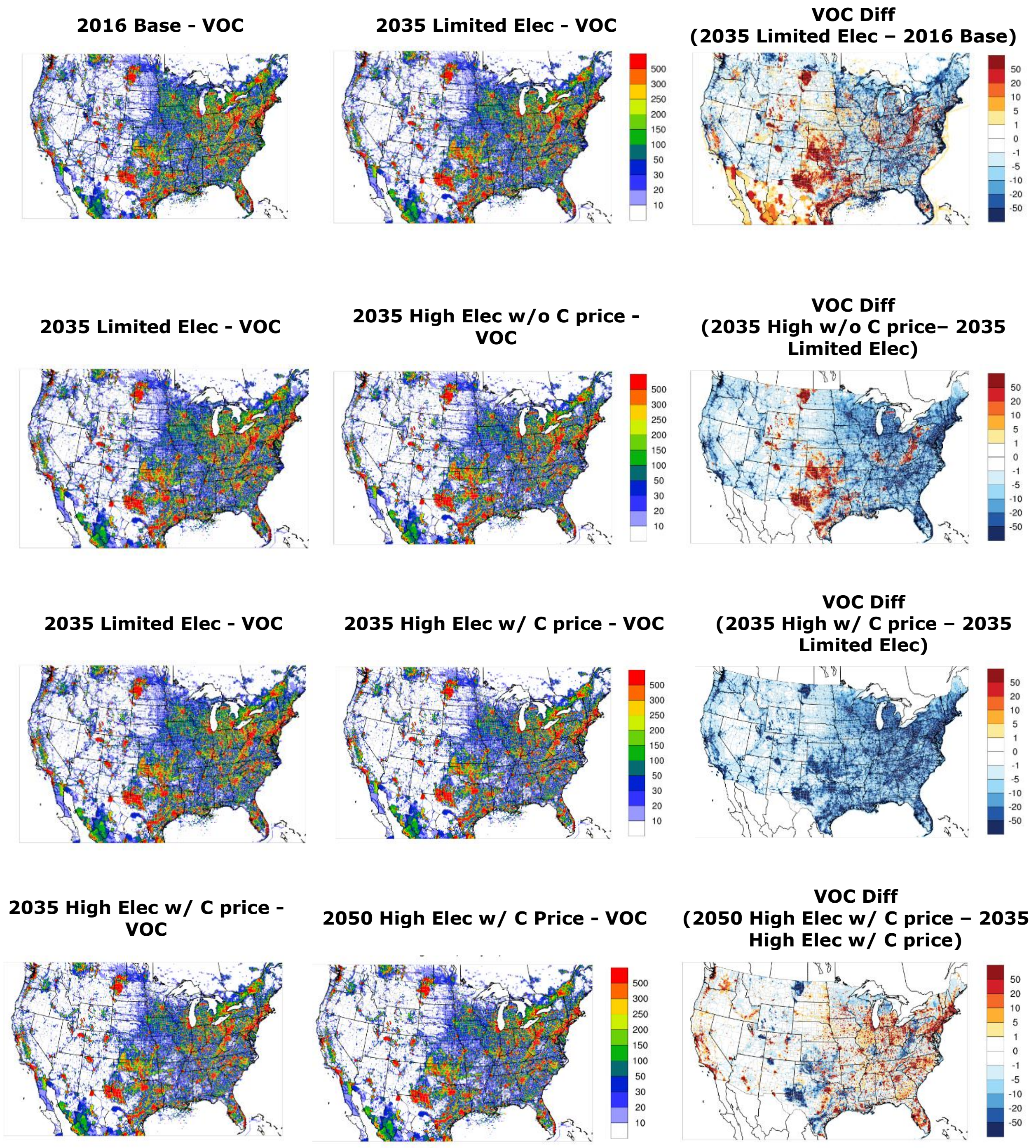

Supplementary Figure 5. Spatial distribution of VOC emissions (tons per year) for the CONUS and emissions difference map between Electrification with Carbon Price scenarios.

VOC emissions are largely driven by changes in four source categories: on-road vehicles, non-road sources, oil and gas activity, and other nonpoint sources. VOC emissions from on-road vehicles and non-road sources decline due to fleet turnover in the 2035 Limited Electrification scenario, but they are offset

by emission increases from oil and gas activity and other nonpoint sources, such as solvent utilization and commercial and residential fuel combustion. High Electrification without Carbon Price can further reduce VOC emissions from on-road and non-road mobile sources due to further levels of electrification. VOC emissions from oil and gas sources decline in the 2035 High Electrification with Carbon Price scenario due to the reduced activity in this sector.

3. Air Quality Modeling Configuration and Initialization

CAMx model configuration: CAMx version 7.0 is a 3-D photochemical transport and dispersion model that has an Eulerian (grid-based) formulation (www.camx.com). The key processes treated by CAMx are emission, transport and dispersion, atmospheric chemical transformation, and deposition to the earth's surface of trace gases and aerosols. CAMx was set up to use Carbon Bond 6 (CB6) gas phase chemistry and other common configurations listed in Supplementary Table 2. The CAMx modeling grids were the 12-km/36-km grids as used in the EPA's 2016 Modeling. The grids are defined using a Lambert-Conformal map projection (Alpha = 33º, Beta = 45º and Gamma = -97º, with a center of X = -97º and Y = 40º) with the southwest corner at (-2412 km, -1620 km). The 12-km domain covers the 48 contiguous states along with southern portions of Canada and northern portions of Mexico (Supplementary Figure 6) with 12-km grid resolution, and has 396 by 246 grid cells and 35 vertical layers.

Supplementary Table 2. Model configurations options for CAMx model

Science Options	2016 Baseline
Version	Version 7.0
Vertical Grid Mesh	35 Layers (no layer collapsing)
Horizontal Grids	12 km nested into a 36 km North American domain
Initial Conditions	10 days full spin-up
Boundary Conditions	2016 GEOS-CHEM day specific
Sub-grid-scale Plumes	No PiG treatment
**Chemistry**	
Gas Phase Chemistry	CB6r4 gas-phase mechanism and CF PM scheme
Aerosol Chemistry	ISORROPIA equilibrium
Cloud Chemistry	RADM-type aqueous chemistry
Meteorological Processor	WRFCAMx v3.4
**Horizontal Transport**	
Eddy Diffusivity Scheme	K-theory with Kh grid size dependence
Source Apportionment	None
**Vertical Transport**	
Eddy Diffusivity Scheme	K-Theory
Vertical Diffusivity Corrections	Kv-patch depending on landuse category up to 100 m and to cloud tops
Planetary Boundary Layer	K-Theory
Deposition Scheme	Zhang. Bi-directional ammonia flux turned off.
**Numerics**	
Gas Phase Chemistry Solver	Euler Backward Iterative (EBI) solver
Horizontal Advection Scheme	Piecewise Parabolic Method (PPM scheme)
Parallelization	OMP-MPI

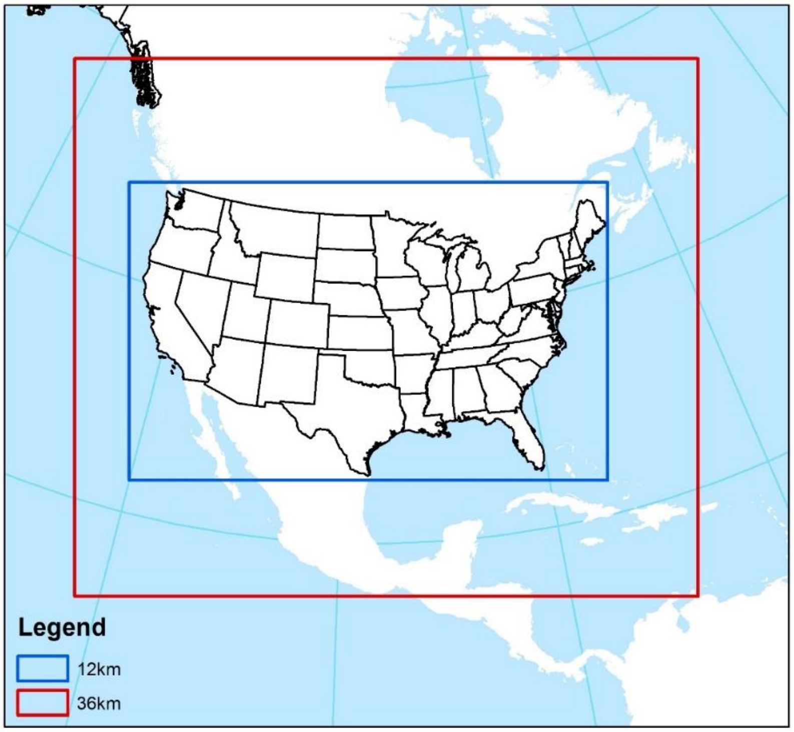

Supplementary Figure 6. The CAMx 12-km and 36-km modeling domains

CAMx initialization*:* Initial and boundary conditions define the air quality at the start of the CAMx simulation and the chemical composition of air transported into the model domain during the simulation via lateral boundaries. The boundary and initial conditions (BC and IC) were obtained from a GEOS-Chem global model simulation performed for 2016. The BCs for future years were assumed to be unchanged from the 2016 baseline BCs. The CAMx model requires inputs for three-dimensional gridded wind, temperature, humidity, cloud/precipitation, and vertical mixing. EPA applied the WRF meteorological model on 36-km and 12-km continental US grids for the year 2016 and reported reasonably good performance. This EPA WRF dataset is used in this study: https://www.epa.gov/sites/default/files/2020-10/documents/met_model_performance-2016_wrf.pdf

EPA evaluated surface winds, temperature, water vapor, and precipitation patterns against observations and found that overall performance was adequate although spatial and temporal variations in performance were noted. Using 12-km grid resolution tended to improve WRF performance over using 36-km grid resolution, and there was a tendency for better WRF performance in the Eastern U.S. than the Western U.S., which can be attributed to the complex terrain of the West. Running WRF and CAMx independently is efficient, because WRF is run only once, but has the limitation of omitting aerosol feedbacks to WRF meteorology although aerosol feedbacks to photolysis reactions are included within CAMx. Uncertainties in PM/O_3 responses introduced by omitting this feedback are likely much smaller than other factors such as the magnitude of the emission changes.

An evaluation of the baseline air quality model performance is important to support conclusions drawn from the future-year scenarios. Ozone performance is evaluated against measurements obtained from the

Clean Air Status and Trends Network (CASTNet) and Air Quality System (AQS). $PM_{2.5}$ is evaluated against measurements obtained from the more rural IMPROVE and more urban Chemical Speciation Monitoring Network (CSN). The CAMx 2016 Baseline simulation shows reasonable performance for both ozone (Supplementary Table 3) and $PM_{2.5}$ (Supplementary Table 4).

Supplementary Table 3. Model performance statistics for MDA8 ozone by season at CASTNet and AQS sites.

	Network	No. of Obs.	Mean$^{(obs)}$	Mean $^{(mod)}$	NMB (%)	NME (%)	FB (%)	FE (%)	Correlation
Spring	AQS	54270	45.8	43.4	-5.2	13.2	-4.6	14.1	0.7
	CASTNET	6354	47.1	43.7	-7.3	13.2	-6.8	14	0.69
Summer	AQS	61028	44.6	44.6	-0.1	14	0.7	14.7	0.79
	CASTNET	6218	45.3	44.9	-0.9	13.9	0.2	14.5	0.76
Fall	AQS	47867	38.7	39.5	1.9	12.8	2.7	13.6	0.79
	CASTNET	6165	39.4	39.6	0.5	12.6	1.6	13.3	0.76
Winter	AQS	31721	35.1	33.8	-3.6	14	-2.2	15	0.71
	CASTNET	6106	36.4	34	-6.6	13.4	-5.7	14.2	0.73
Annual	AQS	194886	42	41.3	-1.7	13.5	-0.8	14.3	0.79
	CASTNET	24843	42.1	40.6	-3.6	13.3	-2.7	14	0.78

Supplementary Table 4. Model performance metrics for PM2.5 and its component species

Species	Season	Network	No. of Obs.	Mean (obs)	Mean (mod)	NMB (%)	NME (%)	FB (%)	FE (%)	Correlation
PM2.5	Spring	IMPROVE	3882	3.6	3.7	3.99	43.2	6.21	40.7	0.53
		CSN	7364	8.0	8.8	10.2	38.8	12	38.5	0.54
	Summer	IMPROVE	3907	5.0	4.4	-13.4	38.5	-18.1	40.3	0.53
		CSN	7055	8.3	9.2	11.3	39.5	13.2	39.1	0.39
	Fall	IMPROVE	3788	3.7	4.3	14.5	45.9	12	42	0.65
		CSN	7297	8.3	10.6	28.3	46.5	27.7	42.6	0.62
	Winter	IMPROVE	3726	2.7	3.6	29.7	60.8	31.8	52.7	0.48
		CSN	7272	9.0	10.7	17.9	46.3	18.4	42.8	0.49
	Annual	IMPROVE	15303	3.8	4.0	5.2	45.3	7.67	43.8	0.54
		CSN	28988	8.4	9.8	17	42.9	17.9	40.8	0.52
PM2.5 SO4	Spring	IMPROVE	3903	0.6	0.7	7.55	38.7	16.6	41.6	0.71
		CASTNET	1015	1.1	1.0	-7.92	21	-6.21	22.7	0.79
		CSN	467	1.0	1.2	21.2	39.4	22.1	36.6	0.61
	Summer	IMPROVE	3943	0.7	0.6	-11.5	46.7	-12.5	52.3	0.64
		CASTNET	1014	1.2	1.0	-16.7	26.9	-24	34.8	0.77
		CSN	404	1.2	1.2	3.58	36.3	0.583	38.5	0.71
	Fall	IMPROVE	3806	0.6	0.7	17.6	48.4	18.7	49	0.76
		CASTNET	1001	1.0	1.0	6.05	25.1	3.89	27.8	0.81
		CSN	330	1.0	1.3	32.2	46.5	32.2	43.1	0.71
	Winter	IMPROVE	3547	0.5	0.6	27.6	51.7	42.1	55.9	0.76
		CASTNET	935	0.9	0.9	-1.74	22	5.42	26.3	0.85
		CSN	401	1.0	1.2	18	44.3	23	41.8	0.53

	Annual	IMPROVE	15199	0.6	0.6	7.57	45.9	15.5	49.6	0.7
		CASTNET	3965	1.0	1.0	-5.85	23.9	-5.47	27.9	0.79
		CSN	1602	1.0	1.2	17.5	41	19	39.7	0.64
PM2.5 NO3	Spring	IMPROVE	3903	0.3	0.2	-30.7	60.7	-40.6	71.3	0.6
		CASTNET	1015	0.6	0.3	-39.4	56	-47.8	69.1	0.58
		CSN	466	0.6	0.6	-0.391	59.9	8.41	55.4	0.56
	Summer	IMPROVE	3943	0.2	0.1	-40.3	70.7	-50.7	77.7	0.27
		CASTNET	1014	0.3	0.2	-46.6	68.1	-58	80.6	0.16
		CSN	404	0.3	0.3	22.7	82.1	11.7	59.8	0.2
	Fall	IMPROVE	3806	0.2	0.2	-7.7	72.7	-12.7	69.7	0.59
		CASTNET	1001	0.5	0.4	-21.2	53.2	-29.6	64.9	0.62
		CSN	329	0.6	0.8	33.4	67.9	37.6	61.2	0.69
	Winter	IMPROVE	3547	0.5	0.4	-28.3	65.5	-17.8	75.8	0.64
		CASTNET	935	1.1	0.7	-36.3	48.3	-37.4	59.2	0.72
		CSN	401	1.5	1.4	-7.93	60.7	8.81	63.1	0.44
	Annual	IMPROVE	15199	0.3	0.2	-26.7	66.5	-31	73.6	0.63
		CASTNET	3965	0.6	0.4	-35.2	53.9	-43.4	68.6	0.69
		CSN	1600	0.7	0.8	3.24	63.6	15.3	59.7	0.57
PM2.5 OA	Spring	IMPROVE	4021	1.8	1.8	-0.846	74.5	27.5	53.9	0.09
		CSN	438	2.3	3.9	71.5	78.7	54.7	60.4	0.71
	Summer	IMPROVE	4026	2.3	2.5	10.6	54	7.45	45.3	0.38
		CSN	400	2.5	4.0	60.1	66.5	40.5	46.5	0.59
	Fall	IMPROVE	3904	1.8	2.2	27.6	61.6	22.8	51.7	0.57
		CSN	331	3.2	4.7	49.2	69.9	52	58.1	0.67
	Winter	IMPROVE	3645	1.1	1.7	61.3	92.5	41.6	66.5	0.35
		CSN	400	2.4	4.5	86.5	97.6	62.8	68.8	0.52
	Annual	IMPROVE	15596	1.7	2.1	19.2	66.9	24.3	54.0	0.11
		CSN	1569	2.5	4.2	66.4	77.9	52.6	58.5	0.58
PM2.5 EC	Spring	IMPROVE	4028	0.1	0.1	-7.3	56.9	1.29	57.8	0.4
		CSN	438	0.5	0.4	-28.5	46.7	-20.1	50.3	0.52
	Summer	IMPROVE	4042	0.1	0.1	-25.6	54.7	-30.9	58.7	0.4
		CSN	400	0.5	0.3	-37.9	49.1	-36.3	55.1	0.36
	Fall	IMPROVE	3905	0.2	0.1	-19.3	49.1	-25	55.9	0.69
		CSN	331	0.6	0.4	-24.1	45.6	-13.9	49.7	0.53
	Winter	IMPROVE	3645	0.1	0.1	3.15	57.4	15.9	63.8	0.63
		CSN	400	0.5	0.5	-12	41	-3.02	45.4	0.64
	Annual	IMPROVE	15620	0.1	0.1	-13.4	54.1	-10.6	58.9	0.51
		CSN	1569	0.5	0.4	-25.3	45.5	-18.5	50.2	0.53
PM2.5 Soil	Spring	IMPROVE	3896	0.6	0.3	-52.7	77.8	-55.8	92.4	0.11
		CSN	446	0.6	0.9	52.9	87.3	45.0	68.0	0.5
	Summer	IMPROVE	3907	0.7	0.3	-54.3	82.6	-49.2	96.1	0.03
		CSN	396	0.8	1.1	31.8	97.8	42.8	79.9	0.09
	Fall	IMPROVE	3781	0.4	0.4	-3.23	97.2	5.22	89.8	0.13
		CSN	330	0.6	1.5	144	166	85.1	95	0.29
	Winter	IMPROVE	3739	0.2	0.2	14.9	107	14	89.8	0.24
		CSN	386	0.4	0.9	125	157	76.5	92.1	0.37
	Annual	IMPROVE	15323	0.5	0.3	-35.2	87	-22	92.1	0.11
		CSN	1558	0.6	1.1	77.5	120	60.7	82.7	0.27

4. Additional Ozone and $PM_{2.5}$ Results

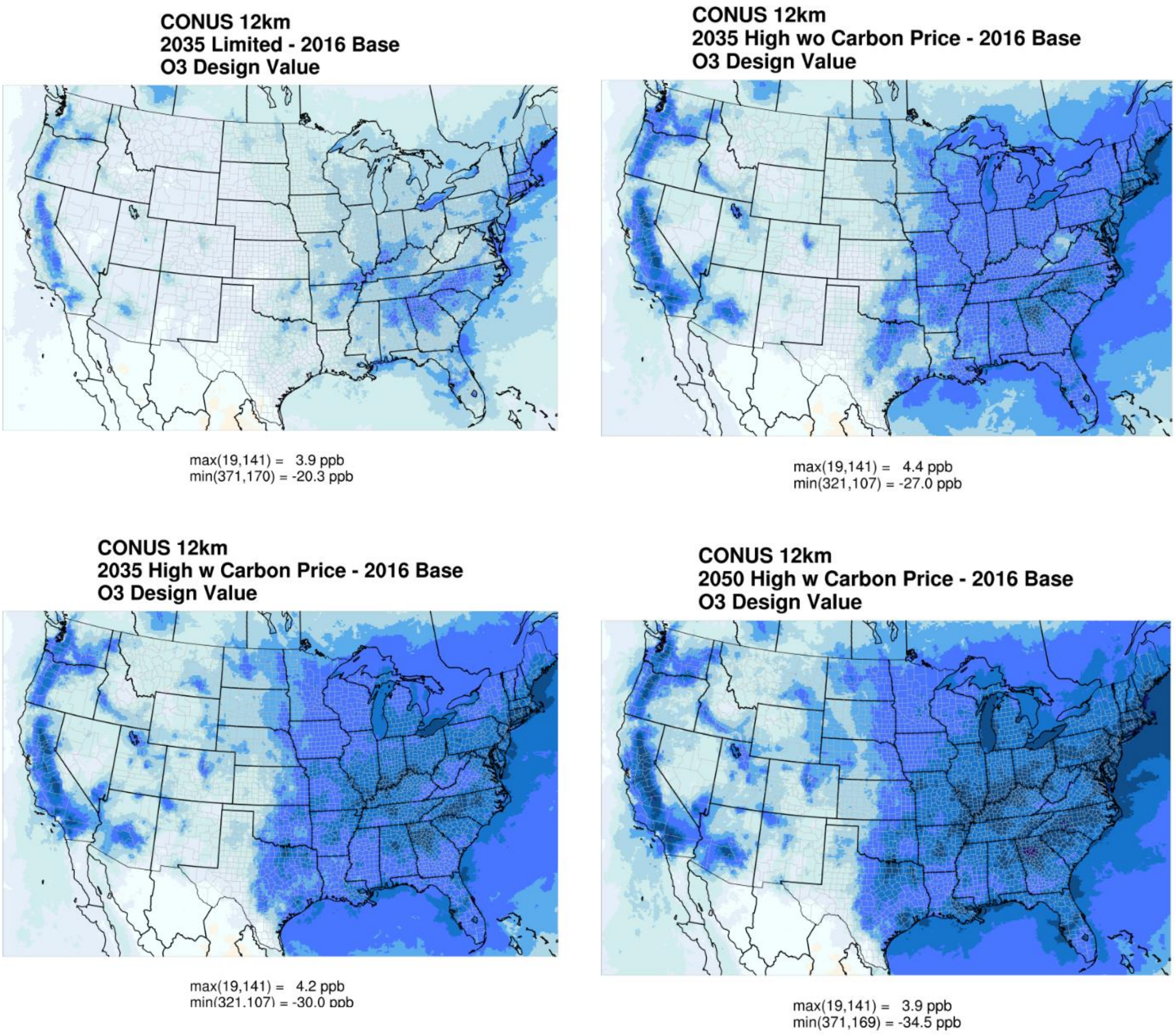

Supplementary Figure 7. Differences in the estimated ozone design values (in parts per billion, ppb) in the CONUS relative to the 2016 baseline. The design value metric under the NAAQS is the fourth highest maximum daily 8-hour average ozone concentrations. Absolute values for these four scenarios and the 2016 baseline are shown in Figure 2 in the main text.

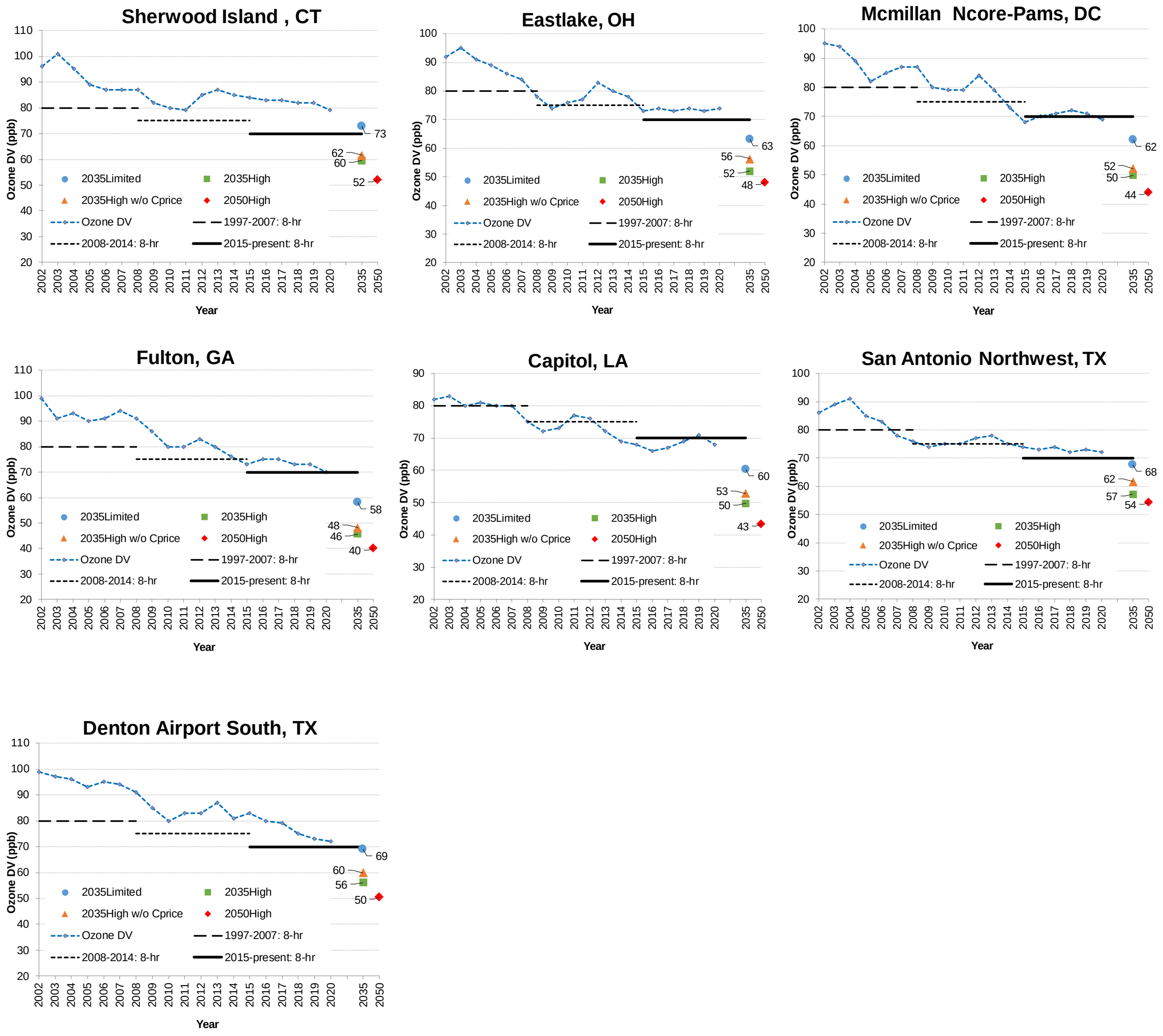

Supplementary Figure 8. Historical ozone design values (DVs) at selected monitors that exceeded the 70 ppb NAAQS in recent years and projected future DVs for electrification scenarios.

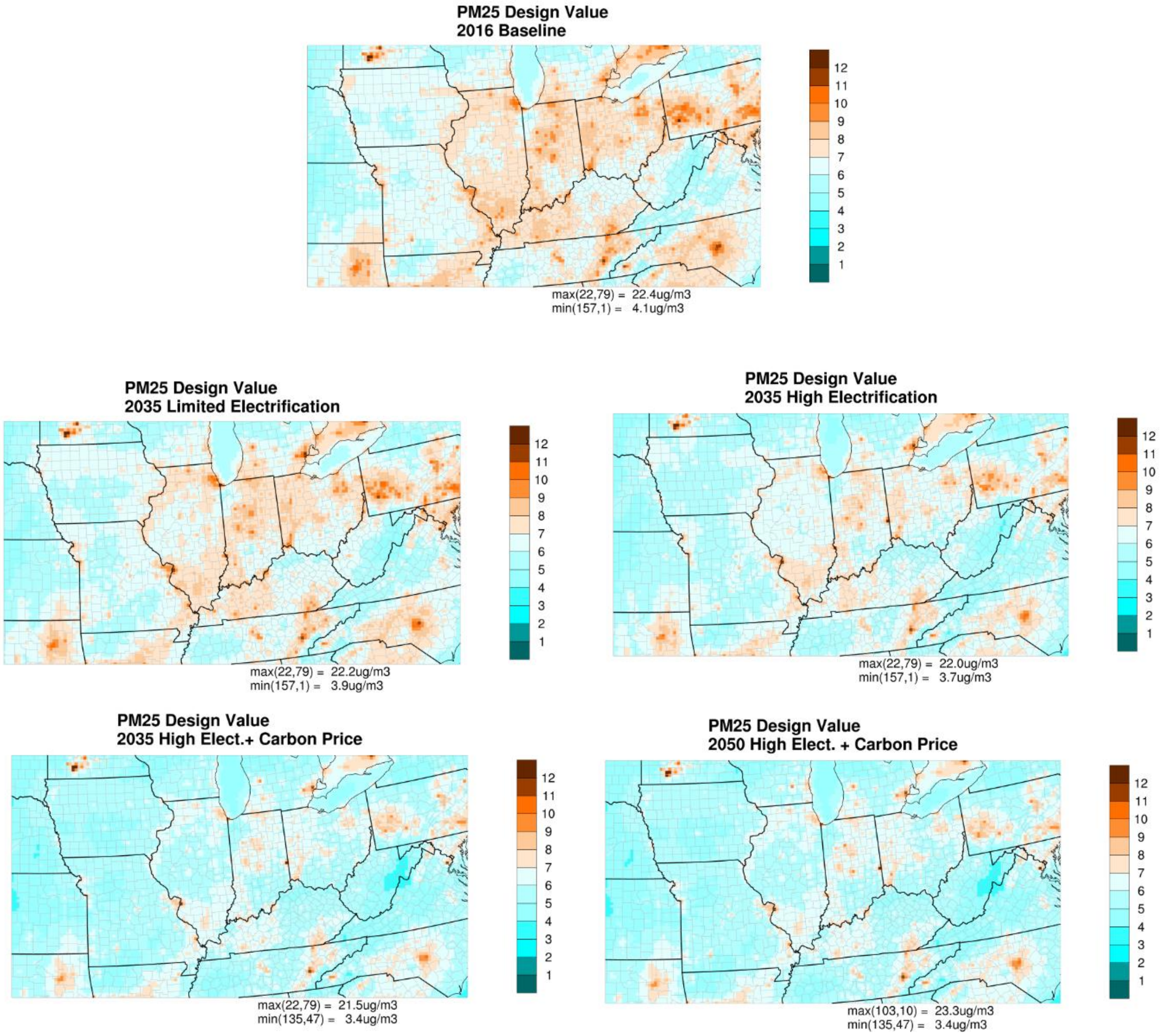

Supplementary Figure 9. Estimated annual $PM_{2.5}$ DVs (μg m^{-3}) in the Midwest U.S.

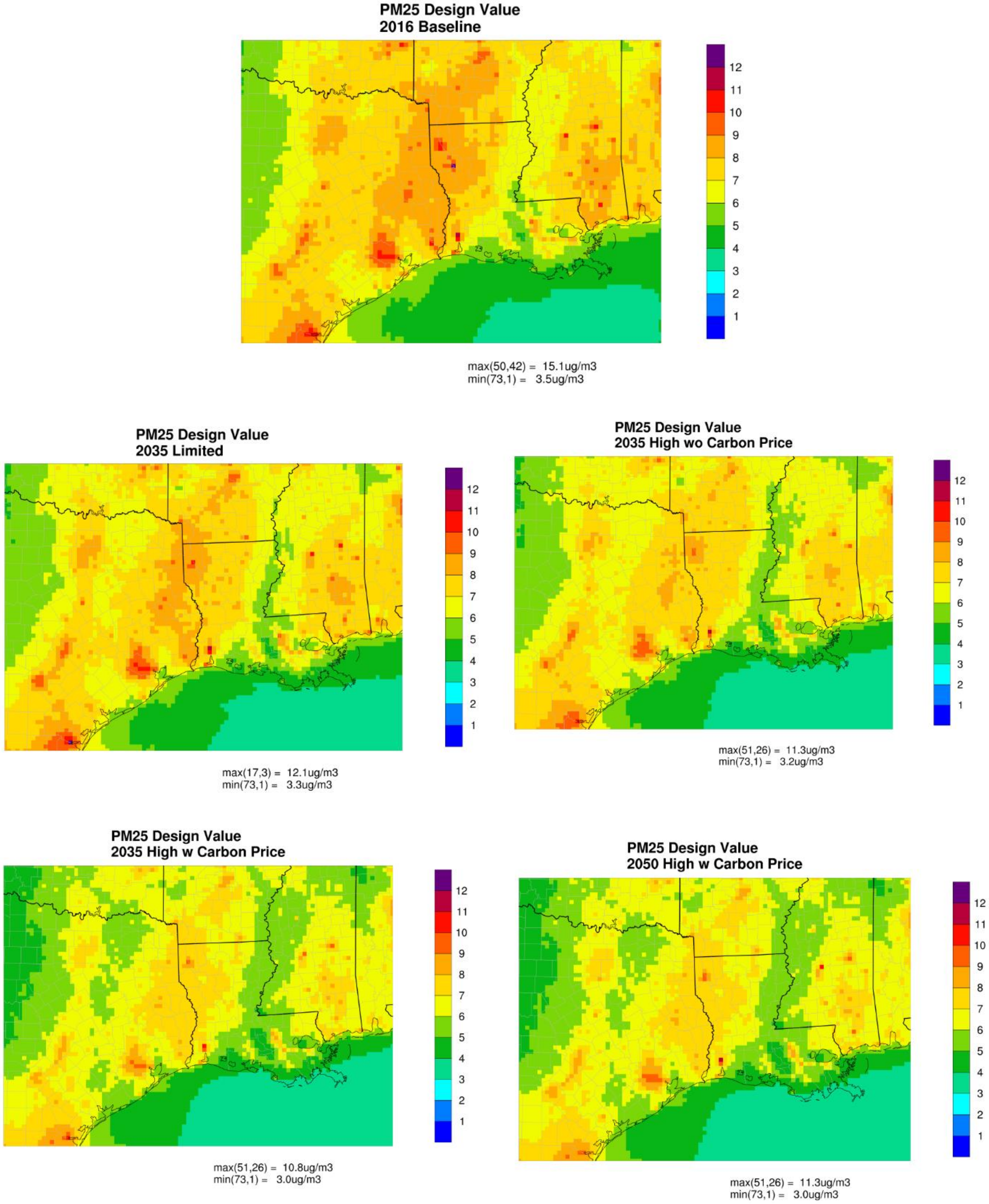

Supplementary Figure 10. Estimated annual $PM_{2.5}$ DVs (µg m^{-3}) for East Texas and Louisiana.

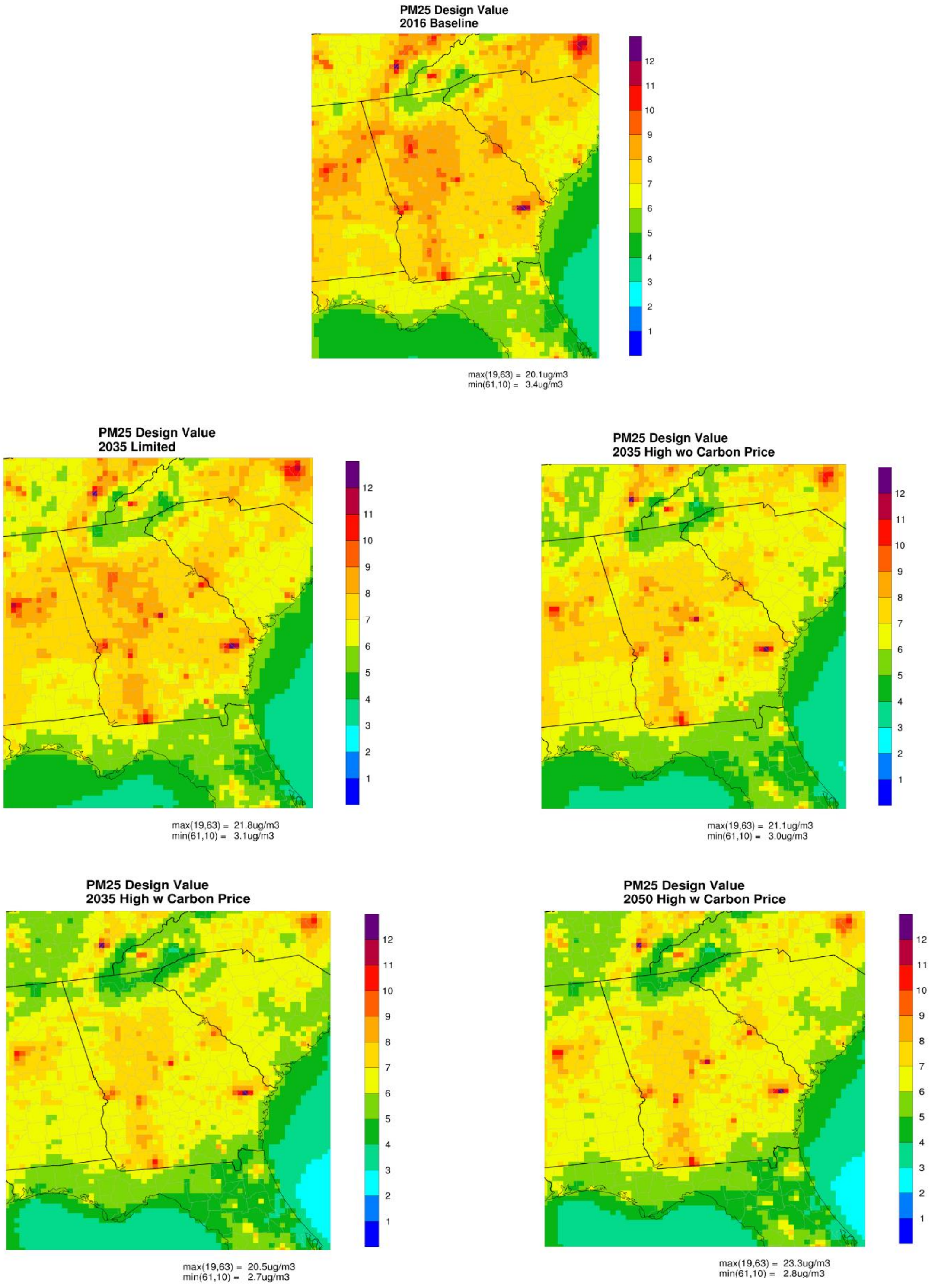

Supplementary Figure 11. Estimated annual $PM_{2.5}$ DVs (µg m^{-3}) for Georgia.

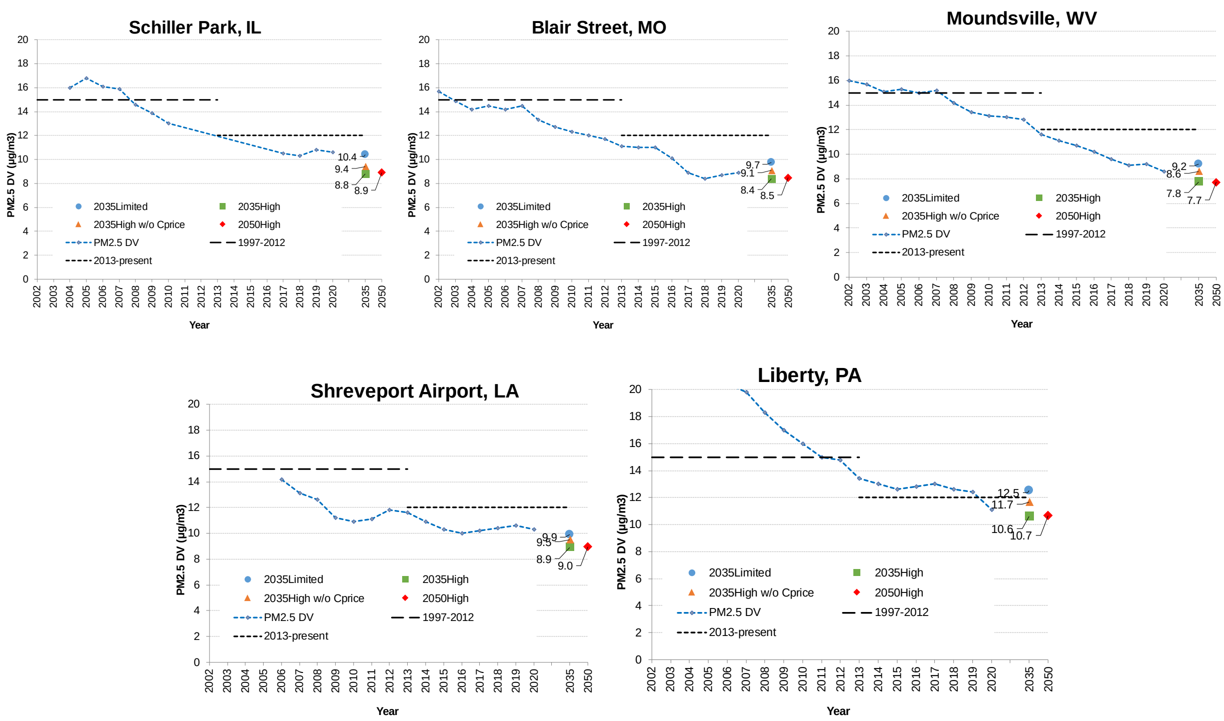

Supplementary Figure 12. Historical $PM_{2.5}$ design values (DVs) at selected monitors.

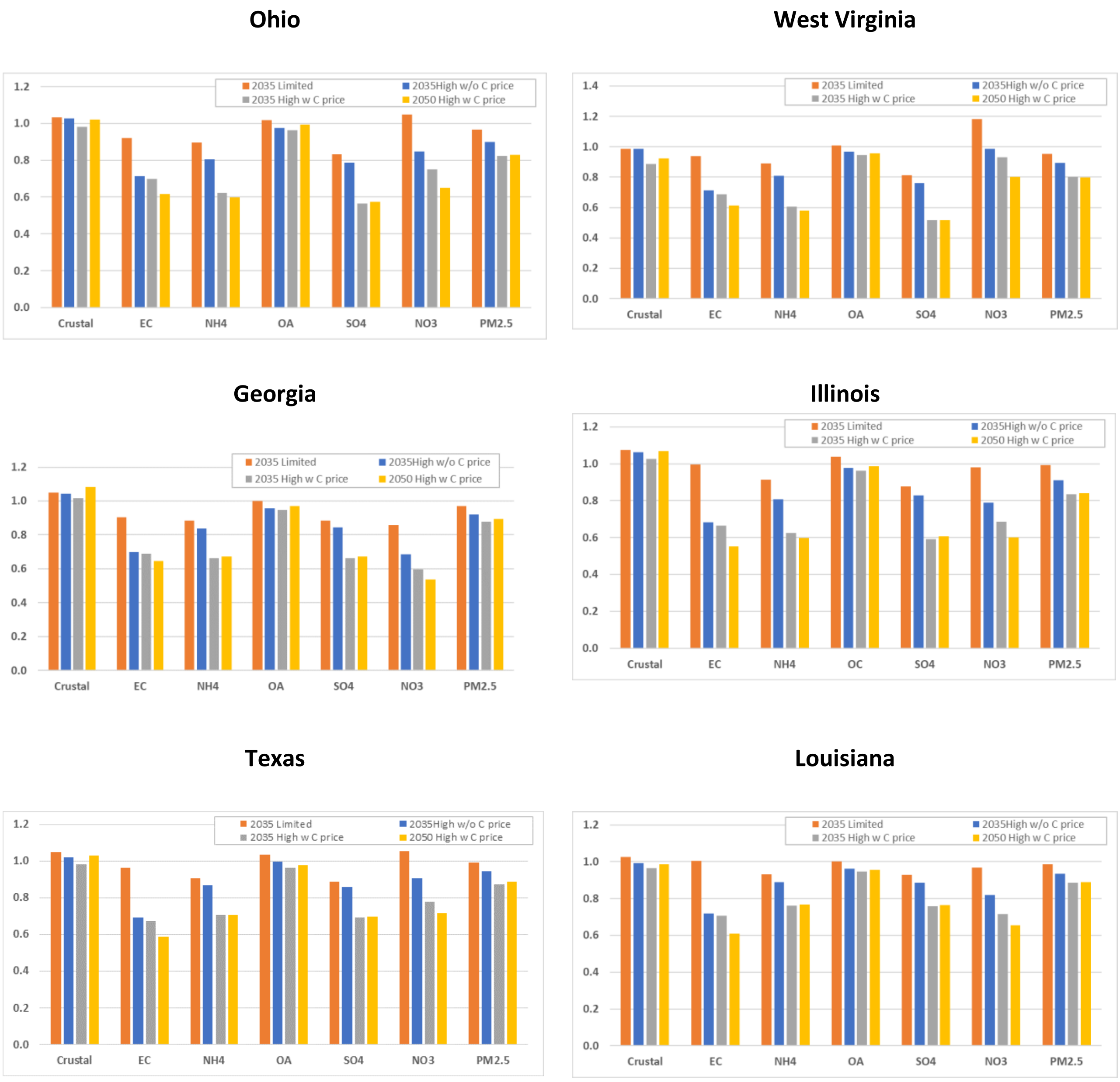

Supplementary Figure 13. Ratio of future year to 2016 annual concentrations averaged across monitors each state. Ratio <1 indicates a decrease of PM component in the future year.

5. Additional Energy System Results

Electrification rates as a share of final energy for these scenarios in 2035 and 2050 generally align with recent economy-wide deep decarbonization and net-zero scenarios (Supplementary Figure 14). Electricity's share of final energy increases in the reference electrification scenario from 21% today to 43% by 2050 in the High Electrification scenario (54% with a carbon tax). Although electricity demand increases with higher end-use electrification, load growth exhibits a broader range. There are three interacting policy-induced effects that lead to differences in electricity demand across models, scenarios, and countries:

- Policies such as CO_2 taxes alter end-use energy prices, which influence fuel demand contingent on policy design and emissions intensities.
- Electricity prices drop relative to fossil fuels due to the greater range of mitigation technologies in the power sector and their relatively low costs, which leads to electrification—the substitution of electricity for fossil fuel use. This leads to higher electricity demand.
- Challenging-to-electrify end uses such as heavy industry and air travel could lead to indirect electrification through electrolytic hydrogen and synthetic hydrocarbon fuels that are derived from electricity. Such indirect electrification can increase load growth, especially since such fuel conversion pathways typically entail low roundtrip efficiencies. However, such alternative fuels face competition from direct electrification, alternative low-emitting fuel pathways (e.g., biofuels), and using fossil fuels with carbon removal.

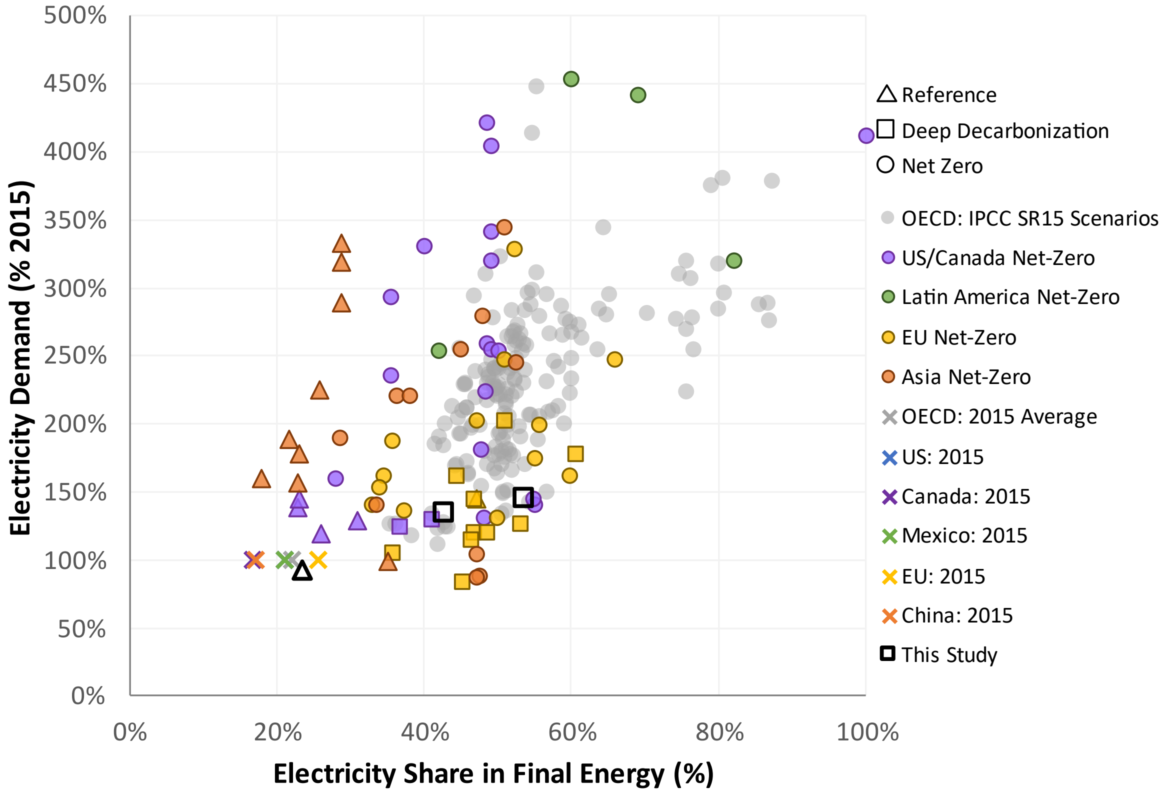

Supplementary Figure 14. Differences in electrification and electricity demand growth across scenarios. Points represent individual scenarios with shapes corresponding to emissions levels (triangle, reference scenario in 2050; square, deep decarbonization in 2050; circle, net zero in 2050), and colors correspond to different analysis regions. Detailed descriptions are provided in Bistline (2021).

Supplementary Figure 15 illustrates how service demand for light-duty vehicles changes over time across different policy scenarios. Electric vehicles (both full battery electric and plug-in hybrid electric) become more competitive over time, and adding a carbon tax increases adoption.

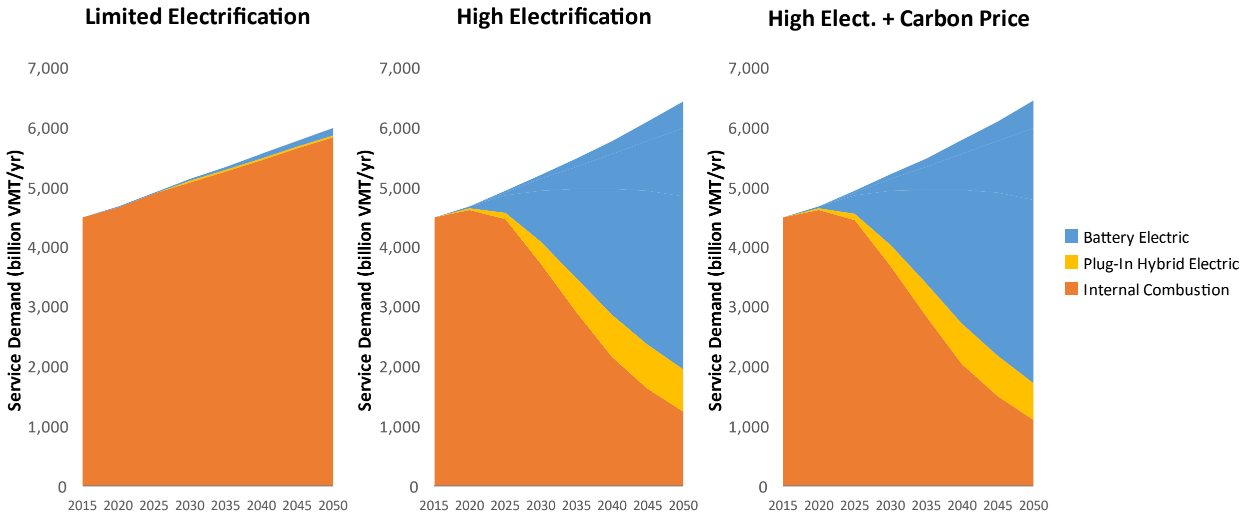

Supplementary Figure 15. Light-duty vehicle service demand by technology across policy scenarios.

Supplementary Figure 16 compares the electric vehicle share of new sales across scenarios in this paper with ones from a recent multi-model study of reaching the 2030 U.S. climate target.[4] This comparison suggests that the High Electrification scenario is more consistent with current market trends that the Limited Electrification scenario, which is used as a baseline to illustrate the air quality impacts of electrification.

[4] Bistline, et al. (2022). "Actions for Reducing U.S. Emissions at Least 50\% by 2030. *Science*, 376(6596):922-924.

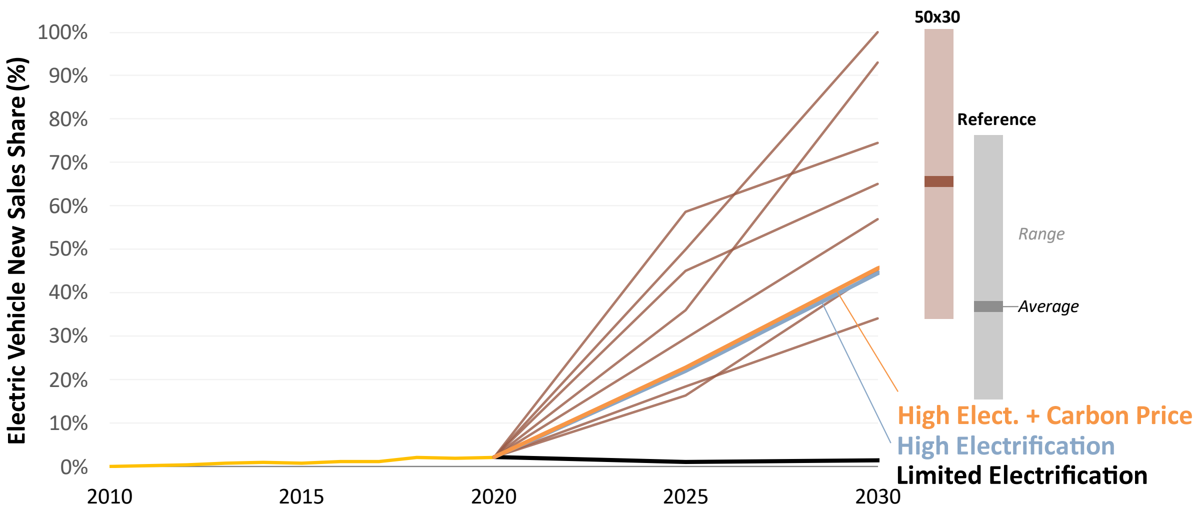

Supplementary Figure 16. Comparison of electric vehicle sales share of U.S. light-duty cars and trucks in the three scenarios in this paper and a recent multi-model comparison of meeting the U.S. 2030 climate target (Bistline, et al., 2022). Red lines show individual model runs to meet the 2030 target, and the ranges on the right show values from these "50x30" scenarios and a current policies reference scenario. Historical values are from the International Energy Agency's "Global EV Outlook 2021."

Electrification shares vary by sector, end-use application, and scenario. Supplementary Figure 17 shows final energy consumption by fuel and end use across model periods.

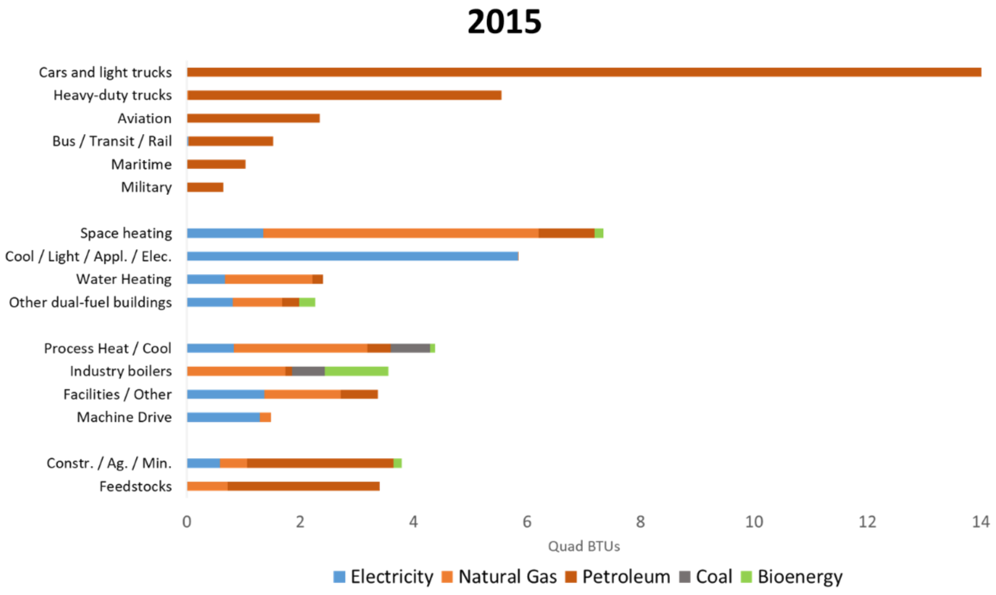

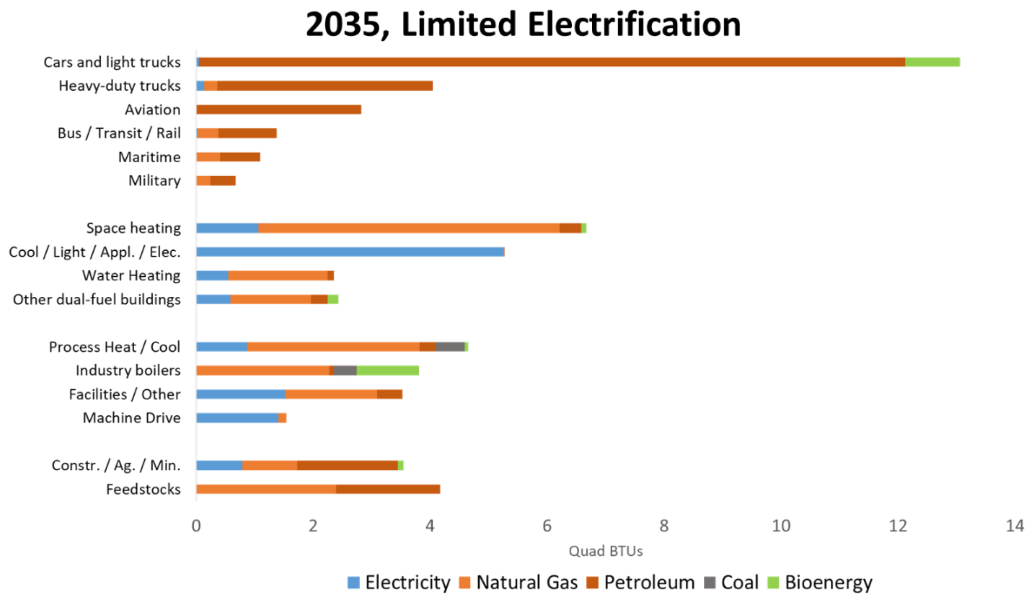

2035, Limited Electrification
Cars and light trucks
Heavy-duty trucks
Aviation
Bus / Transit / Rail
Maritime
Military
Space heating
Cool / Light / Appl. / Elec.
Water Heating
Other dual-fuel buildings
Process Heat / Cool
Industry boilers
Facilities / Other
Machine Drive
Constr. / Ag. / Min.
Feedstocks
0
2
4
6
8
10
12
14
Quad BTUs
Electricity
Natural Gas
Petroleum
Coal
Bioenergy

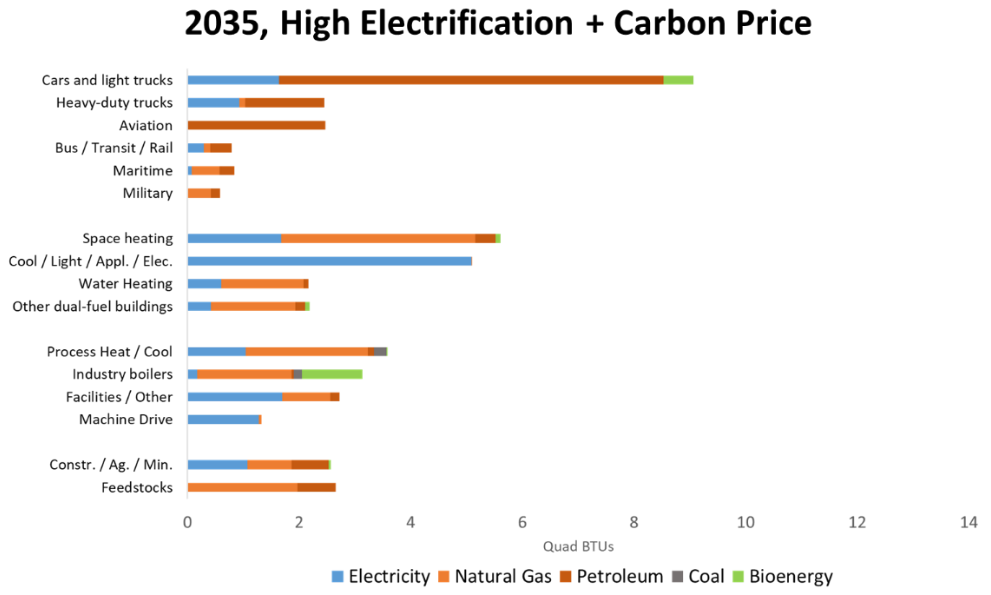

2035, High Electrification + Carbon Price
Cars and light trucks
Heavy-duty trucks
Aviation
Bus / Transit / Rail
Maritime
Military
Space heating
Cool / Light / Appl. / Elec.
Water Heating
Other dual-fuel buildings
Process Heat / Cool
Industry boilers
Facilities / Other
Machine Drive
Constr. / Ag. / Min.
Feedstocks
0
2
4
6
8
10
12
14
Quad BTUs
Electricity
Natural Gas
Petroleum
Coal
Bioenergy

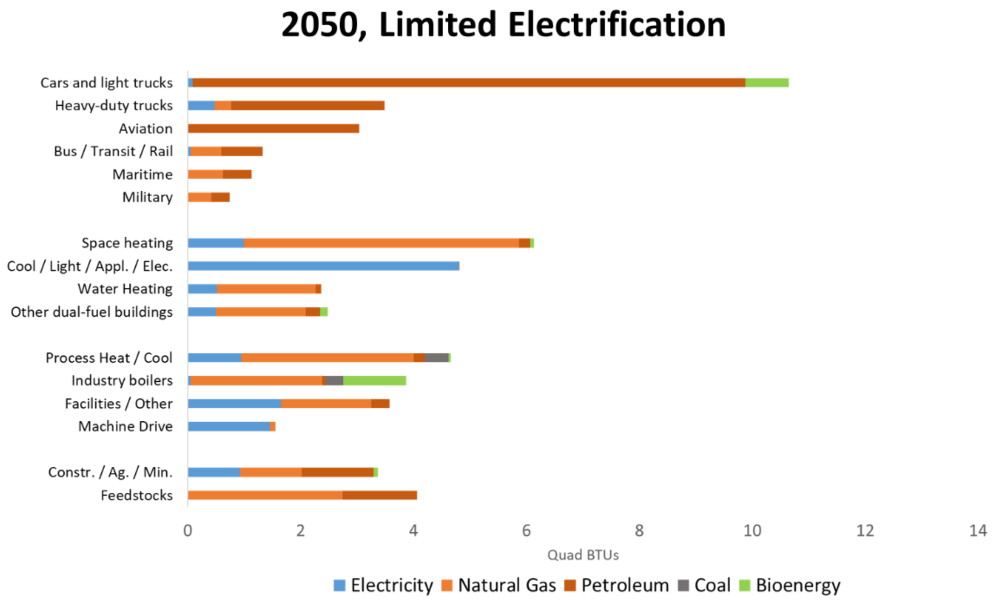

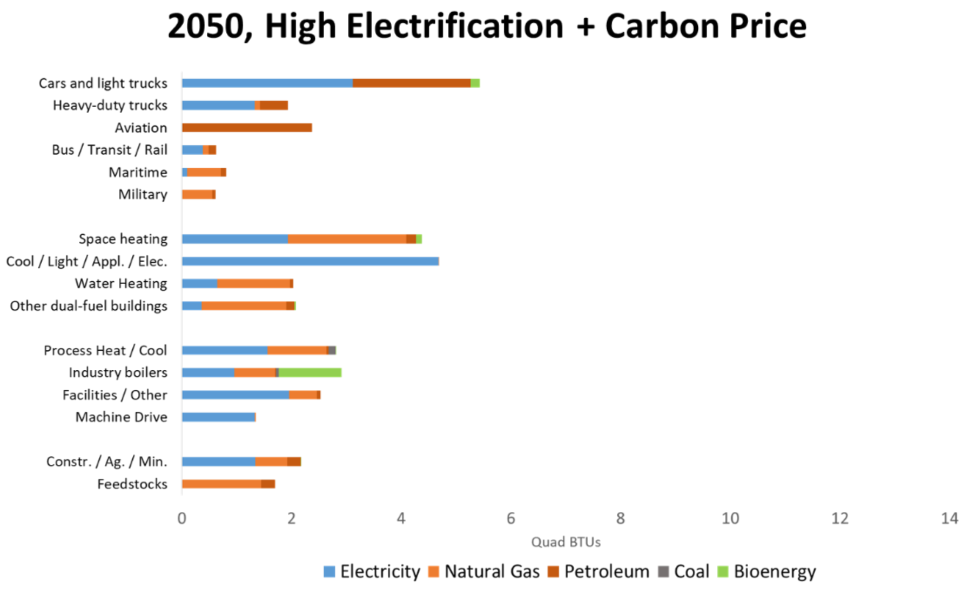

Supplementary Figure 17. Final energy consumption by sector/activity in 2015, 2035, and 2050 in the "Limited Electrification" and "High Electrification + Carbon Price" scenarios.

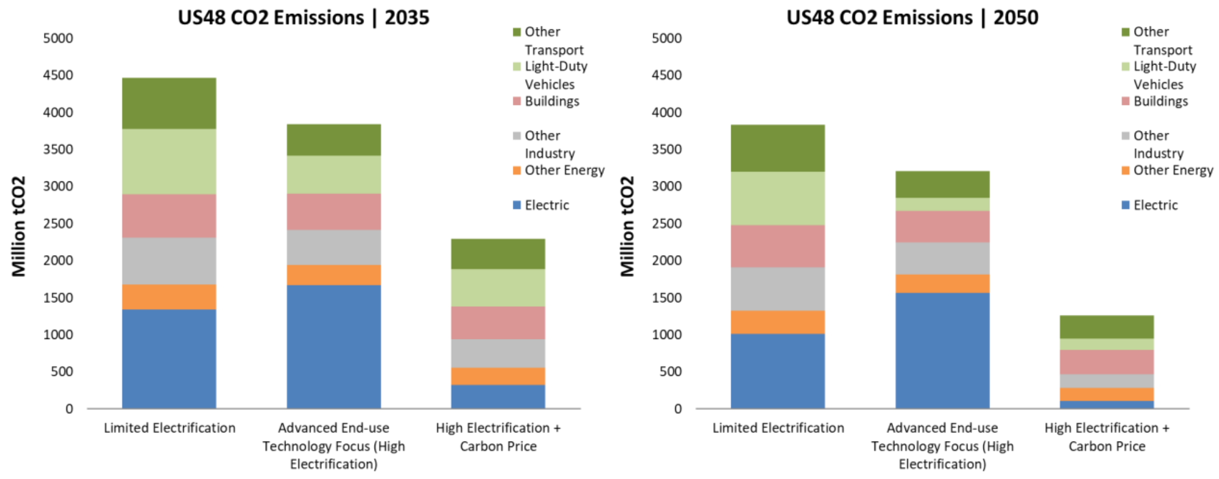

Supplementary Figure 18. Emissions of carbon dioxide by sector in 2035 and 2050 across all scenarios.

Supplementary Figure 19 compares economy-wide CO_2 emissions across scenarios in the analysis with historical emissions and the U.S. Nationally Determined Contribution (NDC) updated in April 2021.

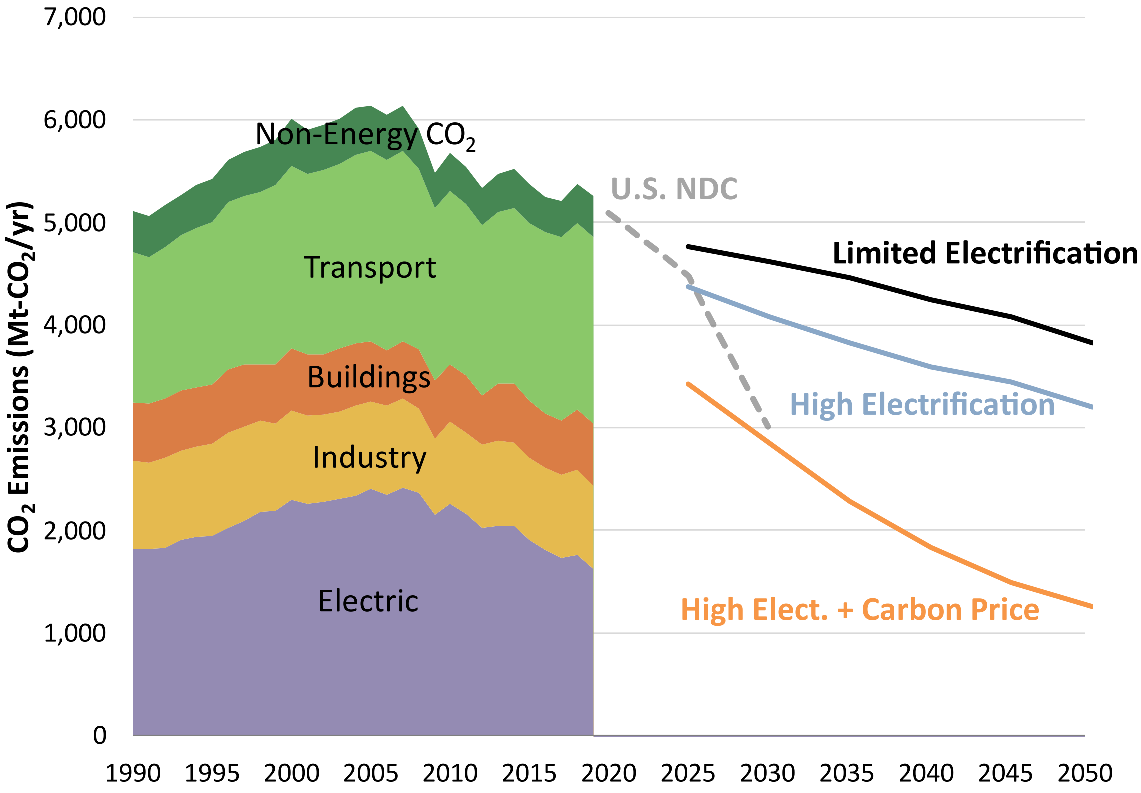

Supplementary Figure 19. Economy-wide CO_2 emissions over time across scenarios. Historical emissions are based on U.S. EPA's "Inventory of U.S. Greenhouse Gas Emissions and Sinks." The dotted line represents the U.S. Nationally Determined Contribution (NDC) updated in April 2021.

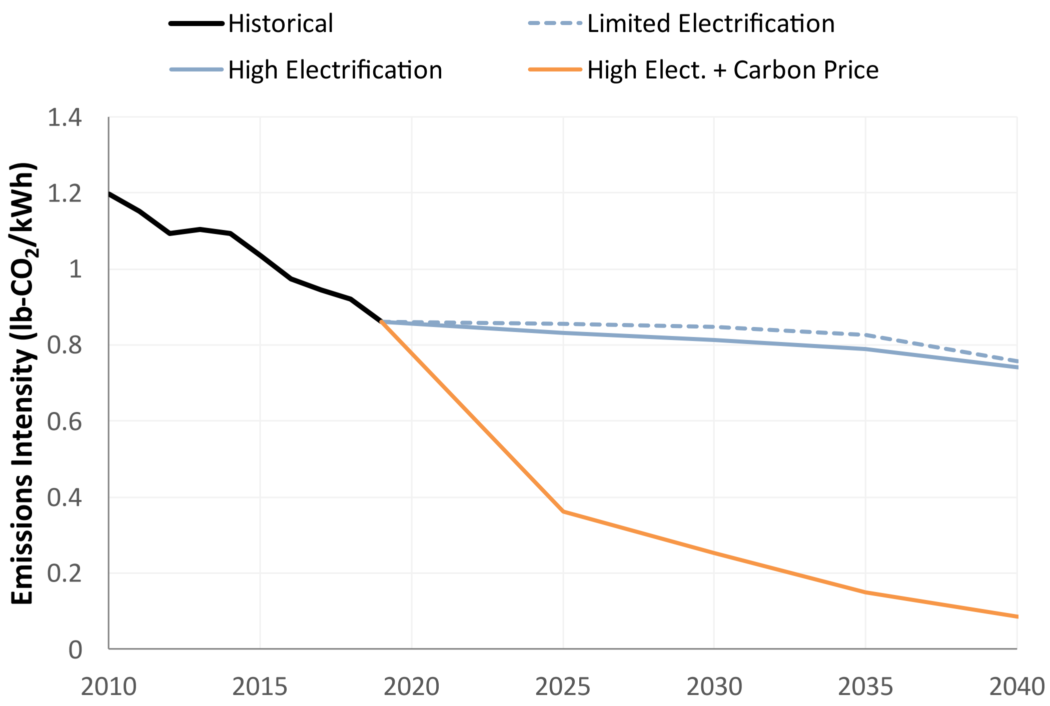

Supplementary Figure 20. Average CO_2 emissions intensity of electricity generation in the electric sector over time and across scenarios.

6. Detailed Scenario Assumptions

Policies in All Scenarios

All scenarios include representations of significant on-the-books federal and state policies and incentives as of June 2021, including:

- State-level renewable portfolio standards, including technology-specific carveouts for solar
- State-level clean electricity standards with state-specific definitions of qualifying resources
- State-level offshore wind mandates (CT, MA, MD, ME, NJ, NY, RI, VA) and energy storage mandates (CA, NJ, NY, VA)
- California AB32, represented as a carbon tax based on projections by the California Air Resources Board
- Regional Greenhouse Gas Initiative (RGGI) cap-and-trade system
- Current Clean Air Act Section 111(b) new source performance standards
- Investment tax credits and production tax credits

The carbon tax scenario layers this economy-wide tax on top of these existing policies. Note that scenarios do not explicitly include the updated U.S. Nationally Determined Contribution pledge to reduce emissions by 50-52% by 2030 from 2005 levels, since formal policies are not yet in place to achieve this target, though the High Electrification with Carbon Price scenario is consistent with this target (Supplementary Figure 19). Scenarios also incorporate announced retirement dates for coal plants.

Limited Electrification Scenario

The “Limited Electrification” scenario is a hypothetical benchmark scenario that can be used to quantify the impact of electrification on outcomes of interest. This scenario is intentionally less optimistic about the speed at which consumers adopt electric end-use technologies across transport, buildings, and industry. This is achieved in the modeling by:

1. Exogenously assuming low EV adoption rates in all future years, as shown in Supplementary Figure 16
2. Imposing a constraint on the electrification of buildings so that shares do not increase beyond today’s levels, which is roughly half of final energy (Supplementary Figure 17, top panel)

These assumptions lead to electricity demand remaining roughly flat over time (Figure 1).

High Electrification Scenarios

The “High Electrification” scenarios use more optimistic assumptions about advanced end-use technologies. Higher electrification in this scenario is driven by:

1. Allowing EVs to deploy endogenously, given continuing trends of falling battery costs (Supplementary Figure 21) and even faster declines in total costs of ownership [1]
2. Accelerating the performance assumptions for heat pumps so that the coefficient of performance improves 10 years faster than in the Limited Electrification scenario (Supplementary Figure 22)
3. Assuming faster turnover rates for the existing equipment stock, tripling the average five-year turnover rate across end-use sector categories from about 10% to 30%, which implies that 30% of existing end-use equipment is eligible to be replaced in a five-year period instead of 10% in the Limited Electrification scenario

Supplementary Figure 21 shows the default electric vehicle battery pack cost assumptions over time and compares these with other projections and historical pack costs.

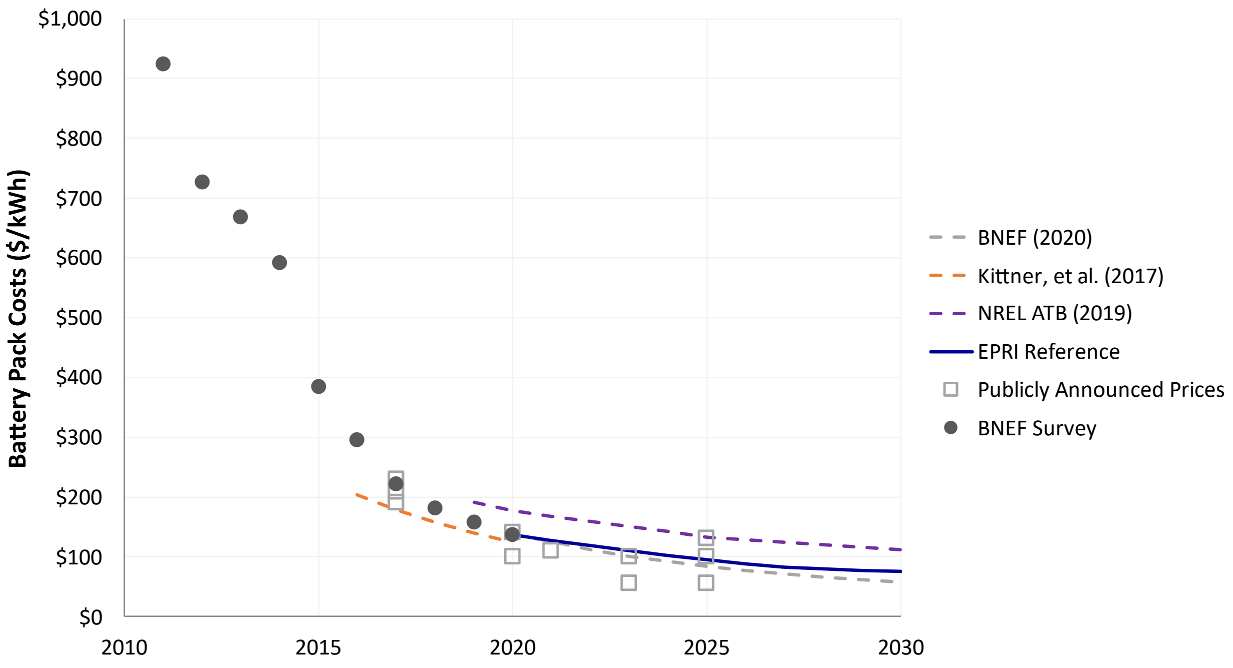

Supplementary Figure 21. Comparison of historical and projected battery pack cost assumptions. Dotted lines show projections from other organizations **[2, 3, 4]**. REGEN battery pack assumptions are shown in solid lines and come from Minear, et al. (2020) **[5]**. BNEF historical battery pack costs, projections, and publicly announced prices come from BNEF (2020) **[4]**.

Supplementary Figure 22 shows the air-source heat pump coefficient of performance assumptions across different outdoor temperature and technology vintages over time. The default US-REGEN assumptions align with the Limited Electrification case in this study. The High Electrification scenarios include accelerations of these performance assumptions by 10 years.

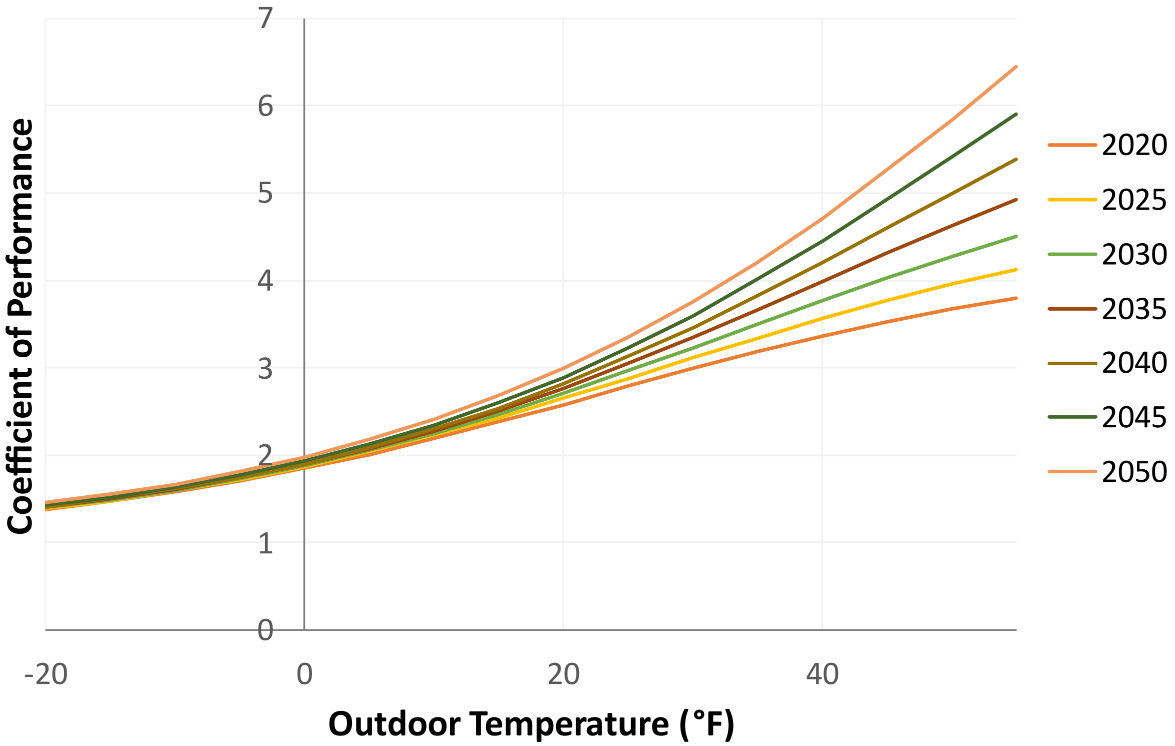

Supplementary Figure 22. Air-source heat pump coefficient of performance (COP) versus outdoor temperature by technology vintage. Values are shown for the Limited Electrification scenario and align with the base US-REGEN assumptions, per Section 3.2.1 of the full model documentation **[6]**.

References

[1] EPRI, "U.S. National Electrification Assessment," EPRI, Palo Alto, CA, 2018.

[2] NREL, "2020 Electricity Annual Technology Baseline (ATB)," Golden, 2020.

[3] N. Kittner, F. Lill and D. Kammen, "Energy Storage Deployment and Innovation for the Clean Energy Transition," *Nature Energy,* vol. 2, 2017.

[4] BNEF, "2020 Lithium-Ion Battery Price Survey," BNEF, 2020.

[5] E. Minear, "Battery Energy Storage Installation Cost Estimation Tool Version 1," EPRI, Palo Alto, CA, 2020.

[6] EPRI, "US-REGEN Model Documentation," EPRI, Palo Alto, CA, 2020.

[7] J. Bistline, C. Roney, D. McCollum and G. Blanford, "Deep Decarbonization Impacts on Electric Load Shapes and Peak Demand," *Environmental Research Letters,* vol. 16, no. 9, 2021.
